# A systems-level analysis of global gene expression in resistant and susceptible *Biomphalaria glabrata* in response to *Schistosoma mansoni* infection

**DOI:** 10.3389/fimmu.2026.1949258

**Published:** 2026-09-15

**Authors:** Abdelmalek Lekired, Coen M. Adema, Si-Ming Zhang

**Affiliations:** Center for Evolutionary and Theoretical Immunology, Department of Biology, University of New Mexico, Albuquerque, NM, United States

**Keywords:** *Biomphalaria glabrata*, constitutive gene expression, gene co-expression network, inducible gene expression, recombinant inbred line, schistosomiasis, transcriptome

## Abstract

**Background:**

The freshwater gastropod *Biomphalaria glabrata* is the major intermediate host of *Schistosoma mansoni*, the causative agent of human schistosomiasis. Elucidating the molecular basis of snail resistance to schistosomes is essential for understanding invertebrate immunity, host–parasite interactions, and schistosomiasis epidemiology.

**Methods:**

Two new *B. glabrata*–*S. mansoni* interaction models were examined: homozygous lines (HZLs) iBS90_R (resistant) and iM line_S (susceptible), and recombinant inbred lines (RILs) RIL18_R (resistant) and RIL108_S (susceptible). Snail transcriptomes were analyzed using RNA sequencing (RNAseq) data from unexposed controls and at 6, 24, and 48 hours post-exposure (hpe) to *S. mansoni*. Differential gene expression and gene co-expression network analyses identified constitutive and schistosome-induced genes and resistance-associated networks.

**Results:**

Each snail line exhibited a different basal transcriptional profile, including immune genes involved in parasite recognition and defense, such as genes encoding lectins, variable immunoglobulin and lectin domain-containing proteins (VIgLs) including fibrinogen-related proteins (FREPs), fibrinogen domain-containing proteins (FReDs), avrRpt2-induced gene products (AIGs), and GTPase immunity-associated proteins (GiMAPs). Resistant lines showed higher constitutive expression of some candidate defense genes, including biomphalysin, *Helix pomatia* agglutinin-related proteins (HREPs), nitric oxide synthase, peptidoglycan recognition protein (PGRP), and aranetoxin. Following infection, susceptible lines mounted weak early responses but underwent extensive transcriptional changes at 48 hpe, including down-regulation of multiple anti-schistosome immune genes. In contrast, resistant lines maintained robust transcriptional activation throughout infection. Although both resistant lines induced similar immune gene families, including biomphalysins, AIGs, thioester-containing proteins (TEPs), and FREPs, they shared few identical differentially expressed genes. Only three genes encoding a perlucin-like C-type lectin (PLL), a multiple epidermal growth factor (EGF)-containing protein, and a GiMAP were consistently up-regulated across all time points in both resistant lines. Co-expression network analysis indicated that resistant snails with distinct genetic backgrounds employ few shared but predominantly lineage-specific immune pathways, highlighting the complexity of host-parasite interactions.

**Conclusions:**

This systems-level comparative analysis of two models with four genetically distinct *B. glabrata* lines provides novel insights into the relationships among genotype, phenotype, and gene regulation involved in the anti-schistosome response. These findings yield a valuable transcriptomic resource for dissecting resistance mechanisms and advancing understanding of invertebrate immunity.

## 1 Introduction

The Neotropical freshwater gastropod *Biomphalaria glabrata* is an important intermediate host of the trematode parasite *Schistosoma mansoni*, a blood fluke that causes human schistosomiasis. Schistosomiasis is one of the most devastating neglected tropical diseases, affecting more than 250 million people worldwide (1, 2). Although the presence of snails is essential for schistosome transmission in endemic regions, transmission ultimately depends on host–parasite compatibility because these snails serve as obligate intermediate hosts in the schistosome life cycle.

A better understanding of the molecular mechanisms that determine whether *B. glabrata* supports or eliminates developing schistosome larvae is crucial for elucidating host–parasite interactions and has important implications for snail-targeted disease control (3–7). The outcome of infection is generally established within three or four days after parasite infection (3, 8, 9). Consequently, considerable effort over the past several decades has focused on characterizing the humoral and cellular immune responses of *B. glabrata* to *S. mansoni* during this critical period. These studies have identified numerous immune-related molecules involved in parasite recognition and elimination, including fibrinogen-related proteins (FREPs) (9–14), superoxide dismutase (SOD) (15, 16), biomphalysins (17, 18), macrophage migration inhibitory factor (MIF) (19), granulin (20, 21), thioester-containing proteins (TEPs) (22, 23), Toll-like receptors (TLRs) and their associated signaling pathways (24–26), and molecules involved in cell-mediated redox responses (27, 28). Recent advances in genomics and other multi-omics technologies have accelerated progress in this field. Additional candidate immune genes and pathways have been uncovered through microarray, RNA sequencing (RNAseq), and proteomic analyses (29–41). Collectively, these studies have significantly advanced our understanding of snail immunobiology and anti-schistosome defense.

To further advance our understanding of the molecular basis of schistosome resistance in *B. glabrata*, we have developed a suite of genetic and genomic resources, including two homozygous lines (HZLs), iBS90 and iM line (42), over one hundred recombinant inbred lines (RILs) (43, 44), and scaffold- and chromosome-level genome assemblies (42, 45). These unique resources provide an unprecedented opportunity to address fundamental questions that cannot be readily resolved using conventional laboratory strains or natural snail populations. In this study, we exploited these genetic resources to ask: whether resistant and susceptible snails (HZL and RIL) differ in their constitutive transcriptional profiles prior to parasite exposure; whether these baseline expression patterns consistently predict infection phenotype across distinct genetic backgrounds; and how transcriptional responses to *S. mansoni* infection differ between resistant and susceptible snails, and between snails with the same phenotype but different genetic backgrounds. We further sought to identify genes and gene regulatory networks that are commonly or uniquely regulated in the two genetic models (HZL and RIL) and to determine, through gene co-expression network analysis, whether parasite-induced transcriptional programs are conserved across resistant genetic backgrounds or instead reflect genotype-specific regulatory mechanisms.

To address these questions, we performed a comprehensive systems-level analysis of genome-wide transcriptomes in four *B. glabrata* genetic lines representing two complementary resistant–susceptible genetic models. By integrating constitutive and schistosome-induced gene expression with gene co-expression network analyses, we identified both conserved and genotype-specific transcriptional programs associated with schistosome resistance. These findings provide new insights into the molecular architecture of host resistance and advance our understanding of transcriptomic regulatory mechanisms underlying anti-schistosome defense in *B. glabrata*.

## 2 Materials and methods

### 2.1 Four genetic lines of *B. glabrata* used in the study

The homozygous *B. glabrata* lines (HZLs) and highly inbred recombinant inbred lines (RILs) were used for this study. The HZL iBS90 and iM lines were generated through 41 and 81 generations of self-fertilization, respectively (42). The RILs were produced by crossing these parental lines followed by 10 generations of selfing (43). The resistant phenotype of iBS90, referred to as iBS90_R, and the susceptible phenotype of the iM line, referred to as iM line_S, were experimentally confirmed using the PR1 strain of *S. mansoni* obtained from the Biomedical Research Institute (BRI), Maryland, USA (42). The phenotypes of the two recombinant inbred lines, RIL18_R (resistant) and RIL108_S (susceptible), were verified using the same parasites (43) (details in review (44)). Each genetic line comprised multiple individuals with identical (HZL) or nearly identical (RIL) genetic backgrounds, and all individuals within a line exhibited the same resistance phenotype.

### 2.2 Exposure of individual snails to *S. mansoni*

Juvenile snails (3–5 mm shell diameter) from four *B. glabrata* genetic lines (iBS90_R, iM line_S, RIL18_R, and RIL108_S) were individually exposed to the PR1 strain of *S. mansoni*. Newly hatched miracidia were obtained from *S. mansoni* eggs, and twenty miracidia were added to each well of a 24-well plate containing a single snail. Exposed snails were collected at 6, 24, and 48 hours post-exposure (hpe), whereas unexposed snails (0 hpe) served as controls (**Figure 1**). The unexposed snails were randomly collected along with snails used for schistosome exposure and maintained for 24 hours under identical controlled experimental conditions (i. e., water, temperature, light, and feeding). For convenience, these unexposed control snails are referred to as 0 hpe snails. Snails used for the study were immediately placed in cryotubes and preserved in liquid nitrogen for RNA extraction.

**Figure 1:**
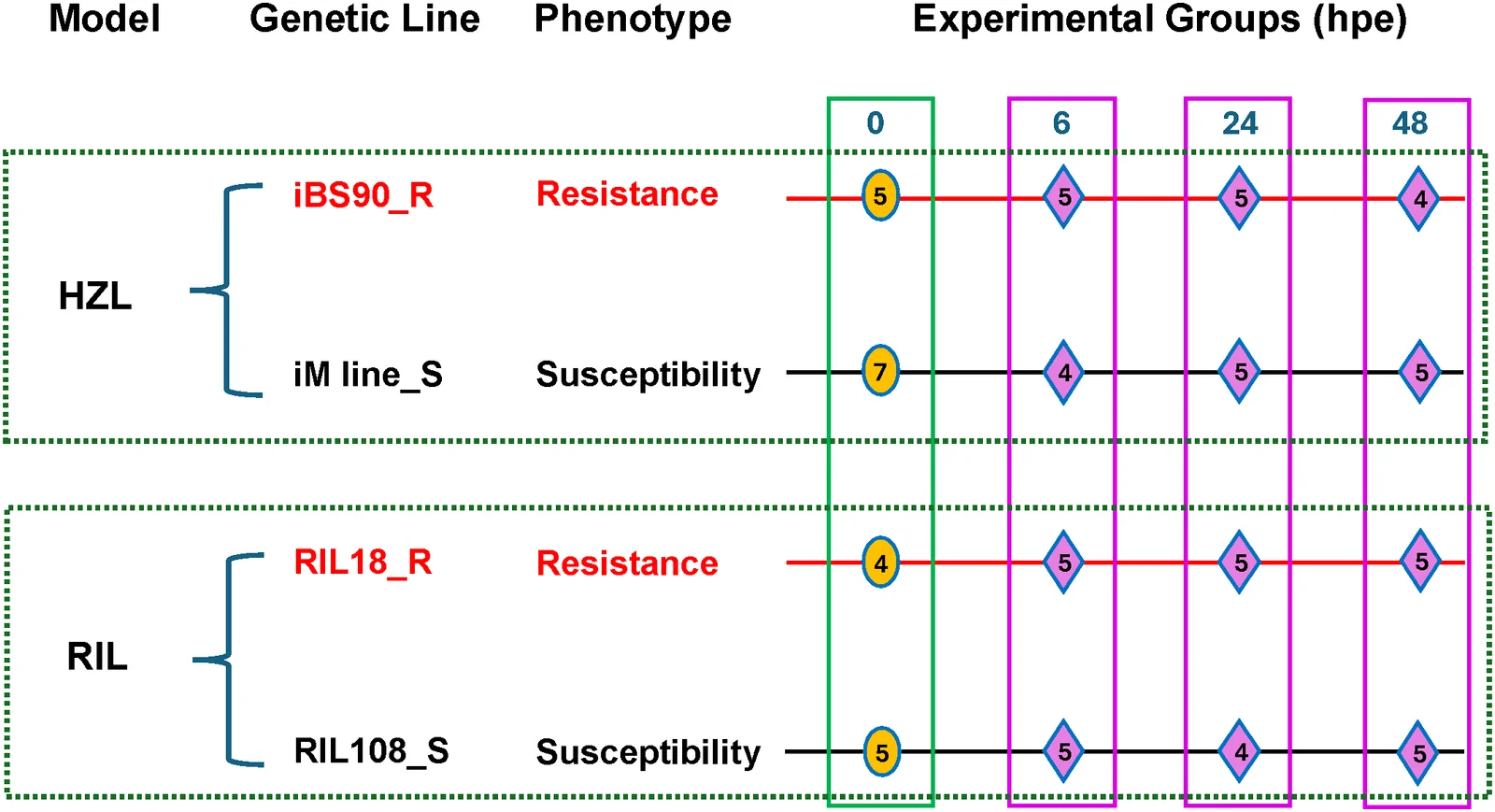
Schematic overview of the experimental design. HZL: homozygous line; RIL: recombinant inbred line; The number in a diamond or oval indicates the sample size at a specific post-exposure time point. hpe: hours post exposure; 0 hpe: control (unexposed snails).

### 2.3 RNA extraction, library preparation, and RNA sequencing

A single intact snail with its shell was transferred to a microcentrifuge tube containing 800 μL of TRIzol™ Reagent (Invitrogen) and homogenized using a sterile plastic pestle. Homogenates were incubated at room temperature for 30 min and centrifuged at 12,000 × g for 3 min to precipitate undigested tissue and shell debris. The supernatant, containing RNA and DNA, was transferred to a fresh tube, and total RNA was purified using the RNA Clean & Concentrator kit (Zymo Research), which includes DNase to remove DNA, according to the manufacturer’s instructions. RNA quality was assessed using an Agilent High Sensitivity RNA TapeStation assay (Agilent Technologies), and RNA concentration was determined using the AccuBlue Broad Range RNA Quantitation Assay (Biotium).

Poly(A)+ mRNA was isolated using the NEBNext^®^ Poly(A) mRNA Magnetic Isolation Module (New England BioLabs). Strand-specific paired-end (2 × 150 bp) sequencing libraries were prepared using the NEBNext^®^ Ultra™ II Directional RNA Library Prep Kit for Illumina (New England BioLabs) and sequenced on an Illumina NovaSeq X Plus platform (Illumina, California, USA). Additional details of library preparation and sequencing have been described previously (46, 47).

### 2.4 Quality control of RNAseq reads

RNA sequencing was performed on individual samples representing four *B. glabrata* lines (iM line_S, iBS90_R, RIL18_R, and RIL108_S). Samples included unexposed controls (0 hpe) and snails collected at 6, 24, and 48 hpe. Raw sequencing read quality was evaluated using FastQC v0.11.9 (48). Adapter removal and quality filtering were performed using Trimmomatic v0.39 (49) with the following parameters: ILLUMINACLIP.fa:2:30:10, AVGQUAL:30, SLIDINGWINDOW:3:20, and MINLEN:36. Potential contaminating reads originating from bacterial, archaeal, fungal, and viral sources were identified and removed using Kraken2 (50). Quality control reports from all samples were summarized using MultiQC (51). Summary statistics for the 78 RNAseq datasets from the individual snails used for the present study are provided in **Supplementary Table 1**.

### 2.5 Differential gene expression analysis

To determine the optimal reference assembly for transcriptome analysis, RNAseq reads were initially aligned against three recently published annotated *B. glabrata* assemblies: iBS90 (GCA_02543165.1), iM line (GCA_025434175.2), and NMRI strain (GCA_947242115.1). Detailed genome information, such as N50 and BUSCO scores for these assemblies, has been previously published (42, 45, 52). The iBS90 assembly yielded the highest mapping rate and was selected as the reference genome for all subsequent analyses (**Supplementary Table 1**). The differences in mapping rates may be attributed to variation in the assembly and annotation of reference genomes and genomic composition among the different lines. RNAseq reads were aligned to the iBS90 genome using HISAT2 (53), and gene-level read counts were generated with featureCounts from the Subread v2.1.1 package (54).

Differential gene expression analysis was performed using DESeq2 v1.42.1 (55). Raw count data were normalized using DESeq2’s size-factor normalization, and differential expression analysis was performed using the normalized count data. For visualization, variance-stabilizing transformation (VST) was applied to the normalized counts, and principal component analysis (PCA) was performed using the VST-transformed data with the plotPCA function in R v4.3.3 (55, 56) (**Supplementary Figure 1**). Unexposed snails were compared for baseline differential expression levels between the resistant and susceptible lines of each model. Differential expression was evaluated separately for the HZL and RIL models by comparing unexposed controls with schistosome-exposed snails at 6, 24, and 48 hpe using the design formula ~snail + hpe + snail:hpe, with susceptible snails set as the reference level for snail and unexposed (control) samples set as the reference level for hpe. All hpe coefficients (6 hpe, 24 hpe, and 48 hpe vs control (0 hpe)) represent the transcriptional responses to schistosome exposure relative to this baseline within the reference line. Comparison pairs were identified using the resultsNames(dds) function in DESeq2. The snail:hpe interaction terms test whether the magnitude of the exposure response differs between the resistant line and the reference susceptible line at each exposure time point, providing a formal statistical test of differential response between the resistant and susceptible lines of each model.

Genes were considered differentially expressed when they exhibited an absolute log_2_ fold change greater than 1 (equivalent to a >2-fold expression difference), a Benjamini–Hochberg false discovery rate (FDR)-adjusted P value <0.01 (57), and a baseMean value ≥10 to exclude genes with very low average normalized expression. This more stringent threshold, compared with the commonly used FDR <0.05, was selected to reduce false-positive results and improve confidence in biologically meaningful expression changes. Shared up-regulated and down-regulated genes among time points were identified and visualized using the ggVennDiagram v1.5.7 package together with ggplot2 in R v4.3.3 (58, 59).

Genome-derived gene IDs were assigned to all differentially expressed genes based on mapping coordinates. Functional annotations were retained from the original BLAST-derived gene descriptions without modification, although multiple genes occasionally shared identical functional descriptors. To improve readability throughout the manuscript, gene identifiers were abbreviated; for example, BgiBS90_020170 is referred to as Bg020170. For each transcript discussed, the abbreviated gene ID and corresponding log_2_ fold change are reported in parentheses.

### 2.6 Weighted gene co-expression network analysis

Variance-stabilizing transformation (VST) values generated by DESeq2 were used as input for weighted gene co-expression network analysis (WGCNA). Separate expression matrices and corresponding trait datasets were constructed for the HZL model (iBS90_R and iM line_S) and RIL model (RIL18_R and RIL108_S), enabling transcriptional responses to be evaluated independently within each genetic background.

Signed co-expression networks were constructed using the WGCNA package in R, which preserves the direction of gene–gene correlations and emphasizes positively correlated gene modules (60). Candidate soft-thresholding powers ranging from 1 to 50 were evaluated using the pickSoftThreshold() function. The lowest power achieving approximate scale-free topology (R² >0.80) while maintaining acceptable network connectivity was selected for network construction. Adjacency matrices were transformed into Topological Overlap Matrices (TOMs), and gene modules were identified by hierarchical clustering of TOM-based dissimilarities followed by dynamic tree cutting with a minimum module size of 30 genes. Modules exhibiting highly correlated eigengenes were merged using the default merge cut height of 0.25.

### 2.7 Trait encoding, gene significance, and hub gene identification

To characterize transcriptional responses specifically associated with schistosome exposure while minimizing constitutive differences between resistant and susceptible genotypes, exposure status was encoded as a binary trait (0/1) within each genetic line. Four genotype-specific schistosome-induced traits were generated in iBS90_R, iM line_S, RIL18_R, and RIL108_S, in which exposed samples were assigned a value of 1 and corresponding unexposed controls were assigned a value of 0.

Module eigengenes were correlated with induction traits using Pearson correlation coefficients. Gene significance (GS) was calculated as the absolute correlation between each gene’s expression profile and the corresponding induction trait. GS values were calculated independently for the HZL and RIL datasets to facilitate direct comparisons of exposure-induced transcriptional responses across genetic backgrounds.

Hub genes within induction-associated modules were identified based on intramodular connectivity (kWithin) and module membership (kME), defined as the correlation between each gene and its corresponding module eigengene. Module-specific gene networks were exported to Cytoscape v3.10.4 (61) using a TOM similarity threshold of 0.02 to visualize network topology and identify highly connected hub genes.

## 3 Results

### 3.1 Strain-specific constitutive differential gene expression in the two models

#### 3.1.1 HZL model: iBS90_R vs. iM line_S

Genes whose basal expression differs between resistant and susceptible lines before infection are referred to as constitutively differentially expressed genes (cDEGs), or baseline differentially expressed genes. In the homozygous line (HZL) model, 769 cDEGs were identified in the resistant iBS90 (iBS90_R) line relative to the susceptible iM line (iM line_S), whereas the susceptible iM line_S exhibited 1,499 cDEGs with constitutively higher expression than iBS90_R (**Figure 2a**). The magnitude of differential expression was substantial for a subset of these genes, with six exhibiting fold changes exceeding 500-fold, including transient receptor potential cation channel subfamily M member 1 (Bg024911; 22,527.7x) (x: fold) and hemagglutinin/amebocyte aggregation factor (Bg010908; 7,834.7x) (**Supplementary Tables 2a, b**).

**Figure 2:**
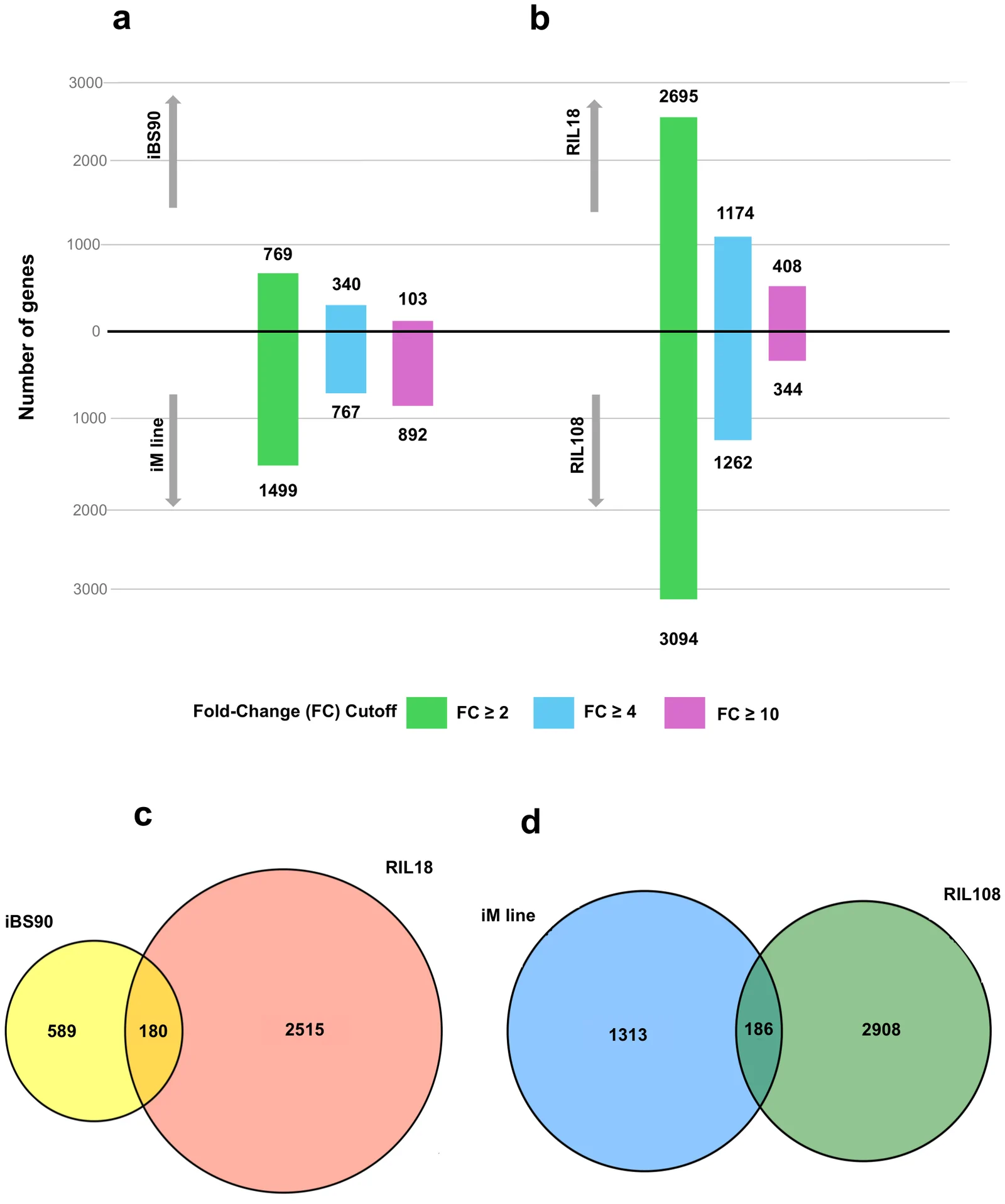
Constitutive differential gene expression (cDGE) in *B. glabrata* models. Constitutively differentially expressed genes (cDGE) in the HZL model (iBS90_R vs. iM line_S) **(a)** and RILs model (RIL18_R vs. RIL108_S) **(b)**. Green represents the total number of DEGs with a fold change (FC) ≥ 2, while blue and purple show subsets with higher FC levels. Shared cDGE in the resistant snail lines (iBS90_R and RIL18_R) **(c)** and the susceptible snail lines (iM line_S and RIL108_S) **(d)**.

Despite the large number of cDEGs exhibiting elevated expression in either homozygous line, the resistant and susceptible snails displayed broadly similar immune functions through distinct sets of genes with comparable functional annotations. Shared immune-related categories included FREPs, fibrinogen domain-containing protein (FReD), TLRs, C-type lectins (including a Perlucin-like lectin, designated as PLL), avrRpt2-induced genes (AIGs), inhibitor of apoptosis proteins (IAPs), GTPase immunity-associated proteins (GiMAPs), mitogen-activated protein kinase kinase (MAPKK), epidermal growth factors (EGF), laccase, latrophilin, and biomphalysin. Immune-related cDEGs uniquely enriched in the susceptible iM line_S included subsets of FREPs, FReDs, biomphalysins, monocyte membrane immune regulator-like (CD108), CD209 (DC-SIGN, a C-type lectin macrophage receptor-like), complement factor-like protein (C1q), galectins 4 and 6, interferon (INF), tumor necrosis factor (TNF) receptors, IL-17, and a macrophage-expressed protein. In contrast, cDEGs of the resistant iBS90_R line exhibited a broader repertoire of variable immunoglobulin and lectin domain-containing proteins (VIgLs), including multiple FREPs, C-type lectin-related protein (CREP), *Helix pomatia* agglutinin-related proteins (HREPs), together with fucolectin, lysozyme, nitric oxide synthase (NOS), and several antimicrobial factors, including peptidoglycan recognition protein (PGRP), aranetoxin, turripeptide, and L-amino acid oxidases (LAAOs).

#### 3.1.2 RIL model: RIL18_R vs. RIL108_S

Compared with the HZL model, the recombinant inbred line (RIL) model displayed a greater number of cDEGs. Resistant RIL18 (RIL_R) and susceptible RIL108 (R108_S) exhibited 2,695 and 3,094 cDEGs, respectively (**Figure 2b**). As observed in the HZL model, both resistant and susceptible lines shared several core immune functions, although these were represented by line-specific cDEG profiles. Common functional categories included GiMAPs, IAPs, FREPs, FReDs, C-type lectins (including PLL), macrophage-expressed-like proteins, fucolectins, galectins, Guadeloupe Resistance Complex (GRC) single-pass transmembrane proteins 2 and 3, TLRs, TEPs, biomphalysin, laccase, achacin, and other LAAOs.

The most highly expressed immune-related cDEGs in RIL108_S included AIGs (Bg030713, 2,593x; Bg030714, 1,042x), additional AIGs (Bg020097, 868x; Bg002694, 225.7x), GiMAPs (Bg002938, 429.3x; Bg020170, 302.8x; Bg034484), a TNF receptor (Bg028943, 232.7x), a C-type lectin (Bg028880, 292x), and multiple FREP12 members (Bg028903, 180.5x; Bg028901, 157.2x; Bg028861, 141x; Bg028890, 1,305x; Bg028927, 129x; Bg028879, 124.8x; Bg028900, 118.4x; Bg028902, 85x). Immune-related cDEGs unique to RIL108_S included another VIgL, C1q-related protein (QREP) (Bg011195, 2.3x), and myeloperoxidase (Bg023790, 11x).

The schistosome-resistant RIL18_R line exhibited a broader diversity of immune-related cDEGs. The most highly expressed immune cDEGs included baculoviral IAP repeat-containing protein (Bg006664, 2,182.9x), tumor necrosis factor ligand family members (Bg011727, 1,949.3x; Bg034072, 1,949.3x), and GiMAPs (Bg016775, 1,691.9x; Bg016776, 1,666.1x; Bg027183, 335.4x). In addition, this resistant line uniquely expressed several FREPs, HREP, GNBP, PGRP, NOS, and multiple antimicrobial peptides (AMPs) (**Supplementary Tables 3a, b**).

#### 3.1.3 Shared cDEGs in snail lines with the same phenotype

Among the 3,464 cDEGs identified across both resistant lines, 180 transcripts representing identical gene sequences were shared between iBS90_R and RIL18_R (**Figure 2c**). Of these, 134 encode proteins with known homologs (**Table 1**). Similarly, among the 4,593 cDEGs identified in the susceptible lines, 186 transcripts were shared (**Figure 2d**), of which 141 have homologous gene products (**Table 2**).

**Table 1:** Shared constitutive expressed genes with known homologs in two resistant lines (iBS90 and RIL18) from two models.

Gene ID	Expression ratio		Gene product	Gene ID	Expression ratio		Gene product
	iBS90	RIL18			iBS90	RIL18	
Bg015137	254.8	96	transient receptor potential cation channel subfamily M member 2, partial	Bg014956	3.7	2.2	RNA-binding protein 7
Bg018513	216.4	252.4	poly [ADP-ribose] polymerase 14	Bg022240	3.7	5.8	inactive pancreatic lipase-related protein 1
Bg017722	156.5	153.4	DNA excision repair protein ERCC-6	Bg024816	3.7	5.3	TLR cluster3 member 20
Bg015138	121.3	91.6	transient receptor potential cation channel subfamily M member 2, partial	Bg032450	3.7	8.2	protoporphyrinogen oxidase-like isoform X2
Bg015136	54.1	87.3	transient receptor potential cation channel subfamily M member 2, partial	Bg030038	3.7	3.4	mitogen-activated protein kinase-binding protein 1
Bg000949	49.9	11.9	Biomphalysin 11, partial	Bg032161	3.7	12.8	THO complex subunit 2
Bg018516	47.3	39.1	poly [ADP-ribose] polymerase 14	Bg033039	3.7	5.5	CAP-Gly domain-containing linker protein 1
Bg032052	46.4	2.9	Nitrogen permease reactivator protein, partial	Bg015041	3.6	3.2	alpha-actinin A
Bg012735	43.7	61.6	Dynein heavy chain 6 axonemal, partial	Bg018439	3.6	2.7	chitin synthase chs-2-like isoform X2
Bg015000	38.4	28.6	flocculation protein FLO11	Bg011040	3.5	2.3	mothers against decapentaplegic 6
Bg033622	34	11.8	CAP-Gly domain-containing linker protein 1	Bg017742	3.5	9.8	transient receptor potential cation channel subfamily M member 1
Bg011643	32.6	19.9	fibrinogen C domain-containing protein 1	Bg024871	3.5	34.5	glutamate-rich protein 6
Bg032977	26.4	18.8	THO complex subunit 2	Bg019130	3.4	3	PE-PGRS family protein
Bg033954	16.9	14.1	multiple epidermal growth factor-like domains protein 6	Bg030112	3.3	2.7	UDP-glucose:glycoprotein glucosyltransferase 1
Bg033027	16.3	47.2	cyclin-dependent kinase 20-like isoform X1	Bg033971	3.3	3.9	zinc finger protein
Bg033413	15.6	46.2	cyclin-dependent kinase 20-like isoform X1	Bg008443	3.2	3.1	glycoprotein-N-acetylgalactosamine 3-beta-galactosyltransferase 1
Bg022389	12.1	8.4	collectin-12	Bg033082	3.2	5.1	E3 ubiquitin-protein ligase RNF168, partial
Bg000979	12	8.4	L-amino-acid oxidase	Bg035197	3.2	3.6	calponiny domain-containing protein
Bg032783	11.9	25.1	metallo-beta-lactamase domain-containing protein 1-like isoform X1	Bg017682	3	3.5	saxiphilin
Bg016006	11.3	3.4	endoglucanase E-4	Bg017695	3	3.4	calcium-binding mitochondrial carrier protein Aralar2
Bg034208	10.6	16.3	transient receptor potential cation channel subfamily M member 1, partial	Bg017715	3	4	peroxisomal trans-2-enoyl-CoA reductase
Bg015094	9.9	8.4	solute carrier family 2 facilitated glucose transporter member 1	Bg018493	3	6.2	hydroxylysine kinase
Bg015206	9.6	7.8	low-density lipoprotein receptor-like isoform X1	Bg018633	3	2.1	stimulated by retinoic acid 6 protein isoform X3
Bg026970	9.4	4.2	ATPase family AAA domain-containing protein 2, partial	Bg019125	3	2.1	kynureninase isoform X2, partial
Bg017685	9.2	170	neurogenic locus Notch protein	Bg024852	3	3.3	homeobox protein Hmx
Bg029144	8.4	32.3	collagen alpha-1(XII) chain	Bg028577	3	2.5	sulfotransferase 1A2
Bg029142	8.2	20.5	collagen alpha-1(XII) chain	Bg034206	3	4.2	UPF0577 protein
Bg001097	7.8	15.8	U3-aranetoxin-Ce1a-like isoform X1	Bg034773	3	4.9	glutamate-rich protein 6
Bg023502	6.7	7.3	protein artemis-like isoform X1	Bg001088	2.9	2.3	alpha-N-acetylgalactosaminidase
Bg029950	6.7	2.7	aminopeptidase N	Bg008744	2.9	2.8	leukemia inhibitory factor receptor-like isoform X2
Bg003648	6.5	5	neuropeptide FF receptor 1	Bg018632	2.9	2.1	BTB/POZ domain-containing protein 6-A
Bg035141	6.5	766.3	sterol regulatory element-binding protein cleavage-activating protein-like isoform X1	Bg022177	2.9	2.5	glycosyltransferase STELLO1 isoform X2, partial
Bg033197	6.2	16.8	microtubule-actin cross-linking factor 1	Bg024345	2.9	8	scavenger receptor cysteine-rich type 1 protein M130, partial
Bg035140	6.1	47.1	calmodulin-like protein 5 isoform X2	Bg030680	2.9	4.2	GTPase IMAP family member 7-like isoform X2
Bg011677	5.9	11.7	multiple epidermal growth factor domains protein 11	Bg033411	2.9	3.2	A disintegrin and metalloproteinase with thrombospondin motifs 7, partial
Bg030071	5.9	4.5	methionine--tRNA ligase mitochondrial	Bg023504	2.8	4.6	scm-like with four MBT domains protein 1
Bg005752	5.7	7	calmodulin-like protein 5 isoform X1	Bg023877	2.8	2.4	S ribosomal protein L28
Bg027182	5.6	174.6	GTPase IMAP family member 4	Bg029667	2.8	2.2	L-threonine dehydratase catabolic TdcB isoform X1
Bg026270	5.5	38.6	denticleless protein	Bg032420	2.8	3.4	forkhead box protein k1, partial
Bg017688	5.4	2.7	neurogenic locus Notch protein, partial	Bg015089	2.7	2.1	histone-lysine N-methyltransferase SETMAR, partial
Bg030044	5.4	3.1	guanine nucleotide exchange factor DBS-like isoform X2, partial	Bg009945	2.7	3.3	dihydropyrimidine dehydrogenase [NADP(+)]
Bg012761	5.3	7.5	E3 ubiquitin-protein ligase RNF168, partial	Bg015080	2.7	2.9	protein rolling stone
Bg022246	5.2	5.5	pancreatic lipase-related protein 2	Bg017696	2.7	3.4	calcium-binding mitochondrial carrier protein Aralar2
Bg005751	5.1	30.9	polcalcin Bra n 2	Bg026944	2.7	2.3	ADAM 17-like protease, partial
Bg014968	5.1	6.6	JmjC domain-containing protein 4	Bg031198	2.7	2.9	acidic repeat-containing protein-like isoform X2
Bg023982	4.9	7.4	von Willebrand factor A domain-containing protein 2	Bg033038	2.7	4.9	hyaluronan-mediated motility receptor
Bg005995	4.8	10.7	temptin	Bg009987	2.6	3	VWFA and cache domain-containing protein 1, partial
Bg034861	4.8	3.1	F-box only protein 39-like isoform X1, partial	Bg017697	2.6	2.4	calcium-binding mitochondrial carrier protein Aralar2
Bg001094	4.7	3.5	U3-aranetoxin-Ce1a	Bg030636	2.6	4.2	glycosyltransferase STELLO2, partial
Bg020202	4.6	3.1	DSC-2	Bg035238	2.6	2.7	calponiny domain-containing protein
Bg024824	4.6	21.6	hydroxyacylsphingosine 1-beta-galactosyltransferase	Bg000917	2.5	3.8	cyclin-G2
Bg006658	4.4	74.6	G-protein coupled receptor GRL101	Bg015347	2.5	2.6	cathepsin-L-like cysteine peptidase
Bg009978	4.4	3.6	cytochrome P450 3a7	Bg018514	2.5	2.8	poly [ADP-ribose] polymerase 14
Bg018519	4.4	3.4	sialin, partial	Bg024338	2.5	6.9	sedoheptulokinase, partial
Bg030179	4.4	14.7	kynurenine formamidase	Bg029937	2.5	3.1	aminopeptidase N, partial
Bg031414	4.4	3.8	quinone oxidoreductase, partial	Bg030971	2.5	2.3	THO complex subunit 2, partial
Bg035193	4.4	4.1	alpha-amidating enzyme precursor 2	Bg032451	2.5	5.4	protoporphyrinogen oxidase
Bg032996	4.3	11.6	methyltransferase-like protein 24	Bg032980	2.5	3.5	mucin-4, partial
Bg032895	4.2	12.5	BgiBFREP21.3	Bg032705	2.5	6	DNA ligase 1-like
Bg001738	4.2	2.2	hemicentin-1	Bg033043	2.5	17.1	short transient receptor potential channel 3
Bg010643	4.2	9.4	DOMON domain-containing protein frrs1L	Bg019836	2.4	2.1	vascular endothelial growth factor receptor 1, partial
Bg024814	4.2	7	TLR cluster3 member 21	Bg026355	2.4	4.6	UBX domain-containing protein 1-A
Bg026350	4.2	24	calponiny domain-containing protein	Bg031473	2.4	4.4	inositol oxygenase, partial
Bg015085	4.1	2.5	guanylate cyclase soluble subunit beta-2-like	Bg018438	2.3	2.4	DNA polymerase lambda
Bg014354	4.1	5	xylan 1 4-beta-xylosidase	Bg021531	2.3	2.5	solute carrier family 17 member 9
Bg024876	4.1	18.9	calponiny domain-containing protein	Bg024304	2.3	10.9	THAP domain-containing protein 1
Bg001092	4	4.6	U3-aranetoxin-ce1a-like isoform x1	Bg024303	2.2	8.2	zinc finger protein 583, partial
Bg018427	4	2.1	semaphorin-1A-like isoform X1	Bg031223	2.2	2.3	Hermansky-Pudlak syndrome 4 protein
Bg032903	4	43.2	hyaluronan-mediated motility receptor	Bg033026	2.2	2.8	A disintegrin and metalloproteinase with thrombospondin motifs 7, partial
Bg026852	3.8	5.8	DNA double-strand break repair Rad50 ATPase	Bg021497	2.2	2.6	proprotein convertase subtilisin/kexin type 5-like
Bg027130	3.8	5.7	deoxyribonuclease-1	Bg000882	2.1	2.8	TBC1 domain family member 19-like isoform X2
Bg014956	3.7	2.2	RNA-binding protein 7	Bg009981	2.1	3.4	trimethylguanosine synthase
Bg022240	3.7	5.8	inactive pancreatic lipase-related protein 1	Bg027070	2.1	3	NADH dehydrogenase [ubiquinone] 1 beta subcomplex subunit 3
Bg024816	3.7	5.3	TLR cluster3 member 20	Bg031234	2.1	2.9	short-chain specific acyl-CoA dehydrogenase mitochondrial
Bg032450	3.7	8.2	protoporphyrinogen oxidase-like isoform X2				

**Table 2:** Shared constitutive expressed genes with known homologs in the susceptible lines (iM line and RIL108) from two models.

Gene ID	Expression ratio		Gene product	Gene ID	Expression ratio		Gene product
	iM line	RIL108			iM line	RIL108	
Bg027168	834.5	55.4	calponiny domain-containing protein	Bg019107	3.6	12.2	muscarinic acetylcholine receptor gar-3
Bg027169	497.2	3.3	trichohyalin	Bg028287	3.6	2	TLR cluster1 member 6
Bg025173	321	21	ATP-dependent DNA helicase HFM1	Bg031441	3.6	2.2	Gametocyte-specific factor 1
Bg027170	174.2	531	tyrosine-protein kinase FRK, partial	Bg005183	3.5	2.5	pancreatic triacylglycerol lipase
Bg011674	157.3	11	scm-like with four MBT domains protein 1	Bg024044	3.4	2.2	zinc finger protein 271-like isoform X1
Bg030311	116.1	409	cytochrome c	Bg016985	3.3	4.5	kDa neurofilament protein
Bg006913	105.2	197.9	fucolectin-7	Bg018261	3.3	6.5	vesicular monoamine transporter
Bg030868	65.5	4.2	CD180 antigen	Bg027202	3.3	6.7	epithelial membrane protein 1, partial
Bg016146	50.3	4.6	Y-box factor	Bg004023	3.2	2.5	signal peptide peptidase-like 2B, partial
Bg017737	39.3	464.7	perlucin-like protein	Bg021431	3.2	5	receptor-type tyrosine-protein phosphatase T-like isoform X1
Bg033694	30.9	2.3	calponiny domain-containing protein	Bg031744	3.2	5.2	aminopeptidase N
Bg033472	28.9	2.8	calponiny domain-containing protein	Bg013730	3.2	3.2	golgin subfamily A member 4
Bg021392	21.8	28.4	neuroligin-2-like isoform X2, partial	Bg014666	3.1	2.7	alpha-(1 3)-fucosyltransferase 7
Bg015596	20.5	9.7	collagen alpha-1(XII) chain	Bg016420	3.1	12.2	tankyrase
Bg030815	17.6	14	oligodendrocyte transcription factor 3	Bg017274	3.1	15.7	DNA-directed RNA polymerase II subunit RPB1
Bg033069	16.5	3.1	alpha-amidating enzyme precursor 1, partial	Bg019203	3.1	9.6	tyrosine-protein phosphatase Lar, partial
Bg033782	16.5	2.5	centromere-associated protein E-like isoform X2	Bg021395	3.1	3.3	ADP-ribosylation factor GTPase-activating protein 1
Bg033679	14.8	2.1	metallo-beta-lactamase domain-containing protein 1-like isoform X1	Bg023603	3.1	4.6	leucine-rich repeat-containing protein 74A
Bg008618	13.7	2.6	trichohyalin	Bg030070	3.1	2.9	transmembrane channel-like protein 3 isoform X2, partial
Bg009936	12.9	29.7	death inducer-obliterator 1	Bg030957	3.1	2.1	arylacetamide deacetylase
Bg033186	11.9	3	peptidyl-glycine alpha-amidating monooxygenase B-like isoform X1, partial	Bg032399	3.1	4.5	ataxin-2
Bg000071	11.7	22.2	C-type lectin domain family 4 member E	Bg002080	3	5.1	E3 ubiquitin-protein ligase arih1
Bg033952	10.2	4.6	zinc finger protein 254	Bg002449	3	3.6	endothelin-converting enzyme 1
Bg014399	10.1	11.1	thyroid hormone receptor alpha-like isoform X2	Bg016206	3	2.5	solute carrier family 22 member 1
Bg024805	9.8	3.5	UDP-glucuronosyltransferase 1-9	Bg017028	3	5	kDa neurofilament protein
Bg017687	9.7	10.1	neurogenic locus Notch protein, partial	Bg021620	3	6.5	RING finger protein unkempt isoform X1
Bg000069	8.8	13.9	C-type lectin domain family 4 member E	Bg001155	2.9	3.9	mitochondria-eating protein
Bg017735	7.8	23.1	mannose-binding protein C	Bg001639	2.9	2.4	inositol polyphosphate multikinase
Bg000169	7.6	4.4	zinc finger protein 708	Bg002793	2.9	3.7	atrial natriuretic peptide receptor 2, partial
Bg024282	7.4	4.6	arylamine N-acetyltransferase 1	Bg029034	2.9	4.3	ADAM family mig-17
Bg006358	7.3	8.3	Biomphalysin 1	Bg031665	2.9	11.2	keratin-associated protein 4-7
Bg023661	7	22.6	macrophage-expressed 1 protein	Bg034849	2.9	3.7	SH3 domain-containing RING finger protein 3
Bg030623	6.9	13.6	mu-type opioid receptor	Bg006936	2.8	3.5	IgGFc-binding protein
Bg001053	6.7	4	vacuolar protein sorting-associated protein 37A	Bg007492	2.8	7.8	FH1/FH2 domain-containing protein 3
Bg001086	6.7	22.7	folate receptor beta	Bg008251	2.8	3.3	arrestin domain-containing protein 3, partial
Bg022210	6.7	4	Pol polyprotein	Bg010147	2.8	2.5	Lin-24 (twenty-four) like
Bg029181	6.7	93.1	basic phospholipase A2 caudoxin	Bg027086	2.8	5.8	RNA polymerase II-associated protein 3
Bg030829	6.6	6.7	nucleolar pre-ribosomal-associated protein 1	Bg029527	2.8	2.2	baculoviral IAP repeat-containing protein 2-like isoform X1
Bg034908	6.5	6	calponiny domain-containing protein	Bg000401	2.7	7.5	D(2) dopamine receptor A isoform X1
Bg014773	6.1	3.3	metabotropic glutamate receptor 3, partial	Bg006845	2.7	3.2	inter-alpha-trypsin inhibitor heavy chain H2
Bg003951	5.6	18.9	rhodopsin	Bg007346	2.7	5.2	retroviral integration site protein Fli-1 isoform X2, partial
Bg031666	5.6	5.3	keratin-associated protein 4-7	Bg015459	2.7	3.8	annexin A7
Bg010554	5.4	7	coumarate--CoA ligase-like 7	Bg025462	2.7	3.1	mucin-like protein
Bg006642	4.9	6.4	ubiquitin thioesterase	Bg014326	2.6	5	retinol dehydrogenase 12
Bg029651	4.9	4.3	antistasin	Bg021915	2.6	5.3	cis-aconitate decarboxylase
Bg011635	4.8	5.2	beta-1 3-galactosyltransferase 1	Bg022296	2.6	3.2	receptor-type tyrosine-protein phosphatase T-like isoform X2
Bg021744	4.8	8.5	platelet endothelial aggregation receptor 1	Bg019257	2.5	3.5	neurotensin receptor type 1
Bg015196	4.7	5.6	alpha-aminoadipic semialdehyde synthase mitochondrial	Bg025184	2.5	2.4	sorting nexin-4-like isoform X3
Bg025789	4.7	94.7	TLR cluster1 member 37	Bg025192	2.5	2.6	mucin-like protein
Bg007750	4.6	2.7	carbohydrate sulfotransferase 15, partial	Bg026866	2.5	3.9	obscurin, partial
Bg009931	4.6	4.7	smg-9 nonsense mediated mRNA decay factor	Bg002698	2.4	3.6	AIG protein
Bg000887	4.5	4.3	sodium/glucose cotransporter 4	Bg013063	2.4	2.5	glucose-6-phosphate translocase
Bg019116	4.5	4.3	carbohydrate sulfotransferase 8	Bg013722	2.4	6.1	ficolin-2, partial
Bg027644	4.5	5	short transient receptor potential channel 3	Bg017521	2.4	3	paired box protein Pax-6, partial
Bg032880	4.5	2.9	trichohyalin	Bg021701	2.4	2.3	fibrinolytic enzyme isozyme C
Bg018503	4.4	2.2	N(4)-(Beta-N-acetylglucosaminyl)-L-asparaginase, partial	Bg023468	2.4	3.9	ankyrin repeat SAM and basic leucine zipper domain-containing protein 1
Bg019489	4.4	2.5	Exostoses (Multiple)-like 3	Bg023694	2.4	2.4	solute carrier family 2 facilitated glucose transporter member 1
Bg001941	4.2	5.5	retinochrome	Bg028831	2.4	2.8	basement membrane-specific heparan sulfate proteoglycan core protein, partial
Bg004503	4.2	4.8	trypsin	Bg031771	2.4	3.3	dipeptidyl peptidase 2
Bg022316	4.2	2.6	platelet endothelial aggregation receptor 1	Bg033876	2.4	3.5	multiple epidermal growth factor-like domains 10, partial
Bg029995	4.2	2.2	myosin-9	Bg019795	2.3	2.2	mitogen-activated protein kinase kinase kinase 3
Bg000950	4.1	6	Biomphalysin 11, partial	Bg021746	2.3	2.7	platelet endothelial aggregation receptor 1
Bg009249	4	22.2	glycine-rich cell wall structural protein-like isoform X1	Bg025031	2.3	4	mucin-4, partial
Bg021380	4	5.8	SET domain-containing protein 9	Bg002498	2.2	3.3	LIM domain-binding protein 3-like isoform X1
Bg029411	4	2.7	dynein heavy chain 6 axonemal-like isoform X3, partial	Bg015709	2.2	2.4	glutamate receptor ionotropic kainate 2
Bg009326	3.9	3.1	GPI-anchored protein PB15E9.01c isoform X1	Bg019072	2.2	2.5	serine-rich adhesin for platelets, partial
Bg013154	3.7	3.8	von Willebrand factor C domain-containing protein 2	Bg021356	2.2	2.8	protein jagged-1b
Bg017686	3.7	20.5	neurogenic locus Notch protein	Bg010855	2.1	2.9	frizzled-5
Bg027094	3.7	5.7	vitellogenin receptor isoform X3, partial	Bg021618	2.1	2.8	phosphatidylinositol 3-kinase regulatory subunit gamma
Bg029637	3.7	4.1	E3 ubiquitin-protein ligase XIAP	Bg023787	2.1	2.4	A-kinase anchor protein 7 isoform gamma
Bg000856	3.6	3.3	glutamate receptor, partial	Bg031184	2	3	serpin B3-like isoform X1, partial
Bg021382	3.6	3.2	DEPTOR protein, partial	Bg031786	2	2.3	mitogen-activated protein kinase kinase kinase 12, partial
Bg021426	3.6	9.5	pathogenesis-related leaf protein 6-like isoform X2				

Two GiMAPs (Bg030680 and Bg027182), poly(ADP-ribose) polymerase (Bg01875), and biomphalysin 11 (Bg000949) were differentially expressed in both resistant lines but not in either susceptible line. Notably, biomphalysin 11 (Bg000949) was shared exclusively by the resistant lines, whereas biomphalysin 1 (Bg006358) was identified as a shared cDEG in both susceptible lines (**Tables 1**, **2**).

Importantly, the 180 shared cDEGs identified independently in the two resistant models (HZL and RIL) showed no overlap with the 186 cDEGs shared between the susceptible lines. Beyond identical shared transcripts, resistant lines also exhibited convergence at the functional level, with non-identical cDEGs encoding proteins with similar immune-related annotations. In particular, both resistant lines showed elevated constitutive expression of several VIgLs (including HREP and specific FREPs), nitric oxide synthase (NOS), PGRP, and the antimicrobial factor aranetoxin (**Supplementary Tables 3a, b**).

### 3.2 Schistosome-induced differential gene expression in the two models

#### 3.2.1 HZL model: iBS90_R vs. iM line_S

Schistosome-induced differentially expressed genes (sDEGs) are defined as genes whose expression levels change significantly following parasite exposure. The schistosome-resistant iBS90_R snails responded to *S. mansoni* infection with substantial schistosome-induced differential gene expression (sDGE) at 6 hpe, comprising 836 up-regulated genes (URGs) and 801 down-regulated genes (DRGs). The number of sDEGs, both URGs and DRGs, increased further at 24 and 48 hpe (**Figures 3**, **4**). Likewise, the number and diversity of immune-related sDEGs increased over time, although only a modest overlap in individual gene sequences was observed between time points (**Figure 5**). The iBS90_R response involved numerous immune gene families, including TLRs, C-type lectins (including PLL), TEPs, FREPs, biomphalysins, NOS, and hemagglutinin (**Table 3**; **Supplementary Tables 4**, **5**). Notably, a transient receptor potential cation channel subfamily M member (TRPM) (Bg024911) consistently exhibited the highest expression level across the three schistosome-induced transcriptomic profiles at all three post-exposure time points in resistant iBS90 snails, with fold changes of 80,471.5x, 50,887.2x, and 26,067.6x compared to unexposed iBS90 snails at 6, 24, and 48 hpe, respectively (**Table 3**; **Supplementary Table 4**).

**Figure 3:**
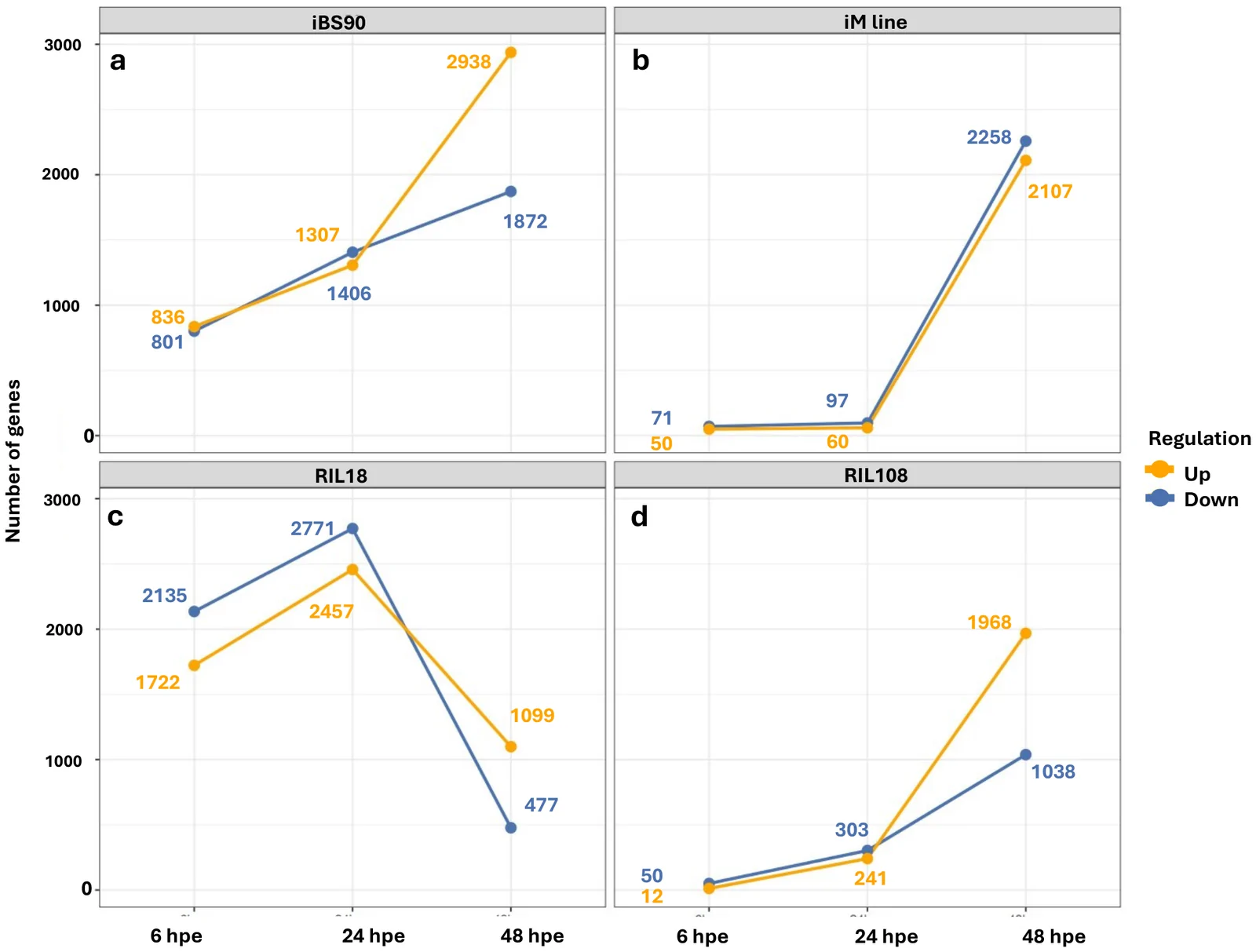
Schistosome-induced differential gene expression (sDGE) in four *B. glabrata* genetic lines. The plot shows the number of up-regulated and down-regulated schistosome-induced differentially expressed genes (sDEGs) at 6, 24, and 48 hpe for iBS90_R **(a)**, iM line_S **(b)**, RIL18_R **(c)**, and RIL108_S **(d)**, respectively.

**Figure 4:**
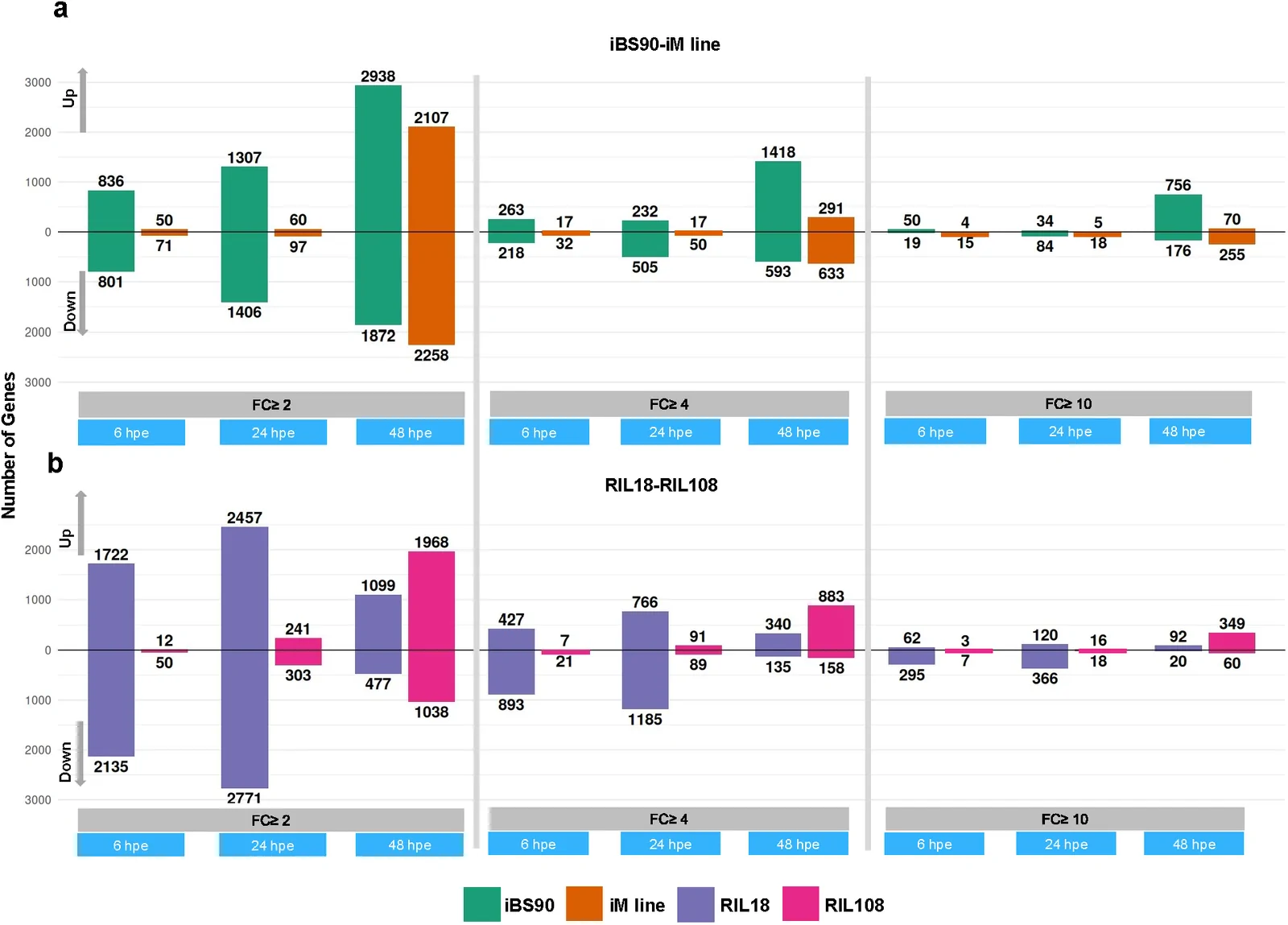
Fold-changes of sDEGs at 6, 24, and 48 hpe in the HZL model (iBS90_R vs iM line_S) **(a)** and RIL model (RIL18_R vs RIL108_S) **(b)**. FC ≥ 2 shows all DEGs, with subsets shown for DEGs with FC ≥ 4 and FC ≥ 10.

**Figure 5:**
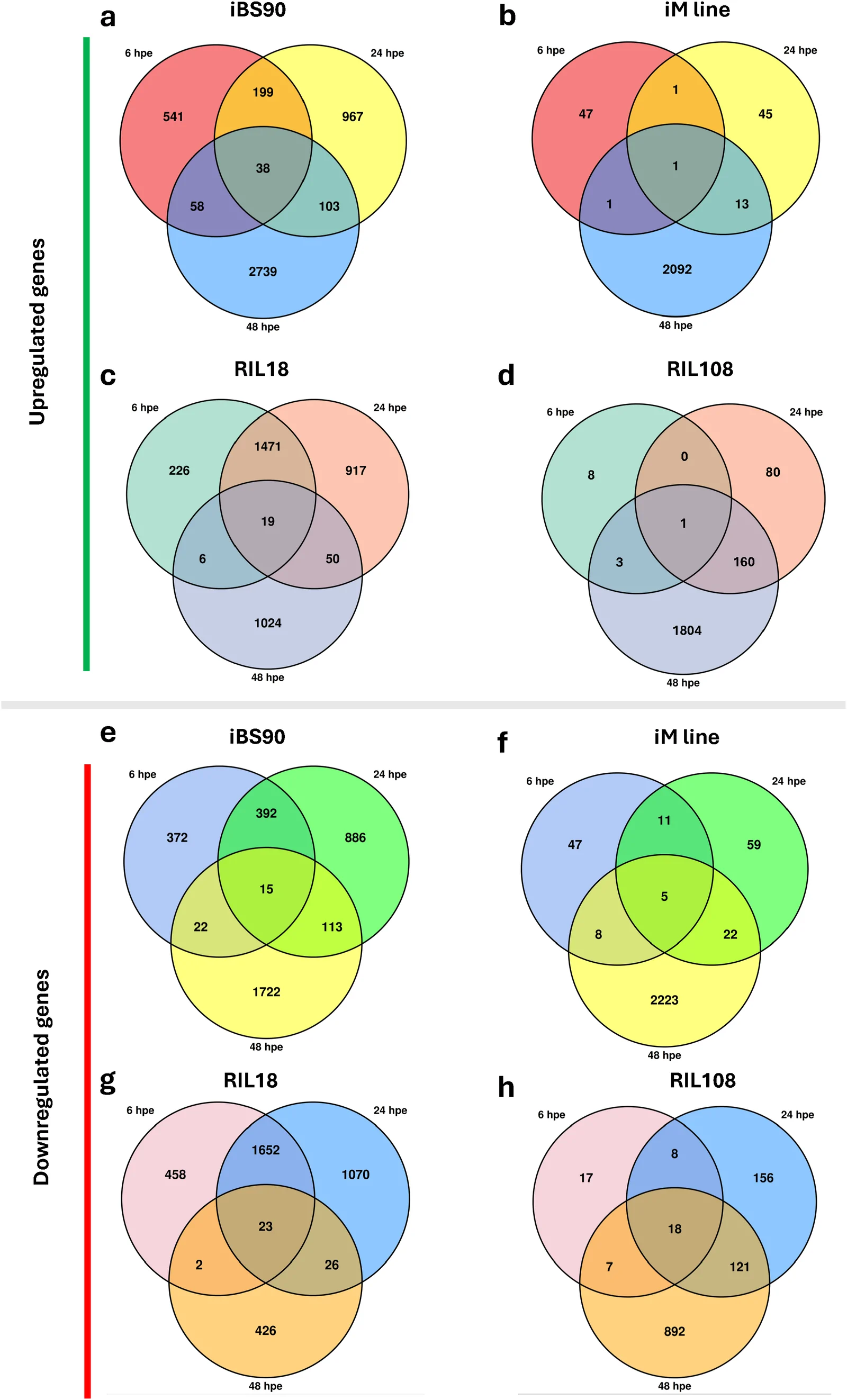
Venn diagrams showing shared up-regulated and down-regulated genes in four snail lines across all time points following exposure to *S. mansoni*. Diagrams **(a–d)** show up-regulated genes in iBS90_R, iM line_S, RIL18_R, and RIL_108_S, respectively, while diagrams **(e–h)** show down-regulated genes in iBS90_R, iM line_S, RIL18_R, and RIL_108_S, respectively. hpe: hours post exposure.

**Table 3:** Shared up- or down-regulated genes with known homologs across three post exposure time points in iBS90 and iM line.

Gene ID	Fold change			Gene product
	6 hpe	24 hpe	48 hpe	
iBS90				
Up-regulated Gene				
Bg024911	80,471.5	50,887.2	26,067.6	Transient receptor potential cation channel subfamily M member 1
Bg006354	12,358.2	8,092.7	7,128.3	D-galacturonic acid binding lectin
Bg033391	30.9	40.3	9.6	THO complex subunit 2
Bg016240	24.6	15.2	27.9	Laminin subunit alpha-like isoform X3, partial
Bg013953	19.9	11.2	45.5	Dynein heavy chain 5 axonemal, partial
Bg017736	15.3	14.6	7.7	Perlucin-like isoform X1, partial
Bg011561	13.8	11.7	10.8	Platelet endothelial aggregation receptor 1
Bg028359	12.6	11.1	13	Calponiny domain-containing protein
Bg011060	8.2	7.2	16.6	Stabilin-2-like isoform X2
Bg011513	7.6	4.8	4.9	Coadhesin, partial
Bg033178	7.1	4.7	2.8	Dynein heavy chain 6 axonemal-like isoform X3, partial
Bg022855	6.8	3	3.6	Monocarboxylate transporter 3
Bg025495	5.8	4.5	13.1	PC-esterase domain-containing protein 1A-like
Bg028884	5.2	3.5	2.5	Bgmfrep12.3
Bg011694	5.1	4.9	4.5	CUB and sushi domain-containing protein, partial
Bg010934	5.1	5.7	7	Properdin
Bg011604	4.7	4	4.8	Hemicentin-1
Bg032268	4.5	3.1	2.4	Melanotransferrin-like isoform X1
Bg031477	4.5	3.6	5.9	A disintegrin and metalloproteinase with thrombospondin motifs 7, partial
Bg035167	4.4	3.8	7.7	ADAM family mig-17, partial
Bg031232	4.3	2.7	2.8	Multiple epidermal growth factor-like domains 10
Bg006763	3.8	3.1	5.2	ADAM family mig-17, partial
Bg030688	3.8	15.2	3.9	Pol polyprotein
Bg014170	3.6	3.2	6.1	F-box only protein 39-like isoform X3, partial
Bg000527	3.2	2.8	2.5	Adhesion G-protein coupled receptor D1, partial
Bg025707	3	4	2.6	G-protein coupled receptor GRL101, partial
Bg006592	3	2.7	4.6	ADAM family mig-17, partial
Bg020546	2.8	2.2	2.5	Cystathionine beta-synthase
Bg013838	2.8	3.1	2.9	T-box transcription factor TBX10, partial
Bg030684	2.6	4.6	3.4	GIMAP protein
Bg017649	2.6	2.5	2.6	Mucin-like protein, partial
Bg026167	2.5	2.6	3.4	Proton myo-inositol cotransporter
Down-regulated Gene				
Bg007613	17.3	25.6	9.6	Leucine-rich repeats and immunoglobulin-like domains protein 2
Bg027500	13.5	5.7	7	CCAAT/enhancer-binding protein alpha-like isoform X2
Bg001297	7.9	9.4	5.6	Ganglioside GM2 activator
Bg008359	5.5	3.4	4.5	Early growth response protein 2
Bg022671	4.7	7.7	37.1	Chlorophyllase-1
Bg020474	3.2	5.7	3.3	Collectin-11
Bg019851	3.2	4	2.7	C-type lectin domain family 3 member A-like isoform X2
Bg025164	3.1	3	2.3	Hypothetical protein bgimline_026730
Bg034976	3	4.4	2.8	Kelch-like protein 12
Bg000498	2.3	4.6	2.1	Glutathione S-transferase 7
Bg010086	2.3	2.1	3.7	Cytochrome P450 3A8
iM line				
Up-regulated Gene				
Bg035293	2.7	3	2.4	Hypothetical protein
Down-regulated Gene				
Bg009108	233.5	177.5	2,756.2	Calponiny domain-containing protein
Bg022342	21.2	28.3	43.7	Multiple epidermal growth factor-like domains protein 11, partial
Bg022339	13.2	7	26.3	Multiple epidermal growth factor-like domains protein 11, partial

In contrast, the *S. mansoni*-susceptible iM line_S exhibited a comparatively modest early response, with only 50 URGs and 71 DRGs at 6 hpe, and 60 URGs and 97 DRGs at 24 hpe. By 48 hpe, however, the response increased markedly to 2,107 URGs and 2,258 DRGs (**Figure 3**). At all time points, subsets of genes displayed varying magnitudes of differential expression, with fold change ranging up to ≥ 10x (**Figure 4**). Few immune genes were represented among the early sDEGs at 6 and 24 hpe. At 48 hpe, a more complex transcriptional profile emerged, characterized by up-regulation of diverse immune factors including LAAOs, lipopolysaccharide-binding bactericidal permeability-increasing protein (LBP/BPI), glutathione S-transferase (GST), granulin, GRC single-pass transmembrane protein 2 genes, laccase, PGRP, and superoxide dismutase (SOD). In contrast, larger numbers of FReDs, FREPs, TEPs, biomphalysins, catalase, TLRs, C-type lectins, and other lectins were down-regulated (**Table 3**; **Supplementary Tables 6**, **7**). Notably, no sDEGs, either among URGs or DRGs, were shared between the resistant BS90_R and susceptible iM line_S snails.

#### 3.2.2 RIL model: RIL18_R vs. RIL108_S

The resistant RIL18_R snails mounted an extensive sDGE response, with 1,722 URGs and 2,135 DRGs at 6 hpe. This increased to 2,457 URGs and 2,771 DRGs at 24 hpe before declining to 1,099 URGs and 477 DRGs at 48 hpe (**Figures 3**, **4**). The response included both up- and down-regulation of a broad range of immune genes. Although relatively few identical transcripts were shared among time points, subsets of genes exhibited progressively stronger differential expression (**Figure 5**). Prominent immune gene families included TLRs, C-type lectins (including PLL), TEPs, FREPs, NOS, and hemagglutinin. At 48 hpe, immune-related DRGs were comparatively few, whereas the URGs included transcripts encoding acanthoscurrin, aranetoxins, PGRPs, AIGs, IAPs, β-1,3-glucan-binding protein, QREPs, CREPs, FREPs, TEPs, biomphalysin, several C-type lectins including PLL, GiMAP, GST, lysozyme, and hemagglutinin/amebocyte aggregation factor (**Table 4**; **Supplementary Tables 8**, **9**).

**Table 4:** Shared up- or down-regulated genes with known homologs across three post exposure time points in RIL18 and RIL108.

Gene ID	Fold change			Gene product
	6 hpe	24 hpe	48 hpe	
RIL18				
Up-regulated gene				
Bg024794	99.4	56.3	16.6	Tolloid-like protein 1
Bg017736	46	205.6	192.7	Perlucin-like isoform X1, partial
Bg024795	39.9	136.8	118.3	Fibroleukin
Bg030693	3.9	11.2	3.3	GIMAP protein
Bg015633	3.1	5.1	2.4	Tyrosine-protein kinase JAK2
Bg026652	3.1	4.4	2.7	GRB2-associated-binding protein 1-like isoform X1
Bg029947	3	3.4	3.1	Aminopeptidase N
Bg029721	3	3.9	2.3	Transcriptional-regulating factor 1
Bg000506	2.9	6.2	2	Allene oxide synthase-lipoxygenase protein
Bg034091	2.7	4.8	2.4	Dentin sialophosphoprotein
Bg002691	2.6	7.8	2.5	GIMAP protein
Bg014034	2.5	3.3	2.8	Serine/threonine-protein kinase prpf4b
Bg034899	2.4	2.8	2.8	Carbonic anhydrase 1, partial
Bg002689	2.2	5	5.7	GIMAP protein
Bg002433	2.2	2.3	2.2	DCN1-like protein 3
Down-regulated gene				
Bg013897	33.3	113.3	5	Tyramine beta-hydroxylase
Bg000151	27.1	19.3	6.4	Coiled-coil domain-containing protein 186
Bg025570	7.4	13.6	3.2	Thyrotropin-releasing hormone receptor
Bg021238	6.8	8.9	10.3	Transient receptor potential cation channel subfamily M member 1
Bg026038	5.6	7.7	3.5	Protein SPT10-like isoform X2
Bg034364	4.8	6.3	4.4	Bone morphogenetic protein 1, partial
Bg030951	2.9	4	2.2	Feeding circuit activating peptides-like isoform X4
Bg013762	2.8	3.2	4.2	Extensin-2-like isoform X2
Bg009339	2.8	5.5	2.8	Coumarate--coa ligase
Bg010055	2.7	5.9	2.4	Pre-rrna-processing protein FHL1
Bg017997	2.5	2.9	2	Alkylglycerol monooxygenase, partial
Bg007237	2.4	7.1	4.4	Beta-1 3-glucan-binding protein
Bg020473	2.4	2.7	3.5	Hemicentin-1, partial
Bg014531	2.4	3.3	2.9	Prqfvamide
Bg026550	2.4	2.2	2.2	Enterin
Bg017236	2.1	4.9	2.5	Frizzled-4
Bg020055	2.1	4	3	Jerky protein, partial
Bg032263	2	2.3	2.6	Hydroxytryptamine receptor 7
RIL108				
Up-regulated gene				
Bg001108	15.3	6.7	4.3	Dermatopontin
Down-regulated gene				
Bg009881	16.3	9.6	20.9	Sodium-coupled monocarboxylate transporter 2
Bg008359	8.5	9	5.4	Early growth response protein 2
Bg027500	7.5	10.8	6.5	CCAAT/enhancer-binding protein alpha-like isoform X2
Bg005551	5.2	6	3.3	Cysteine dioxygenase 1
Bg014796	4.2	3.8	8.2	Solute carrier family 26 member 6
Bg016098	3.4	3.7	2.1	myosin-16-like isoform X1
Bg013552	3.3	5.4	5.6	Galactosylceramide sulfotransferase
Bg025716	3.2	3.3	2.1	Abelson tyrosine-protein kinase 2
Bg003965	3.2	3.2	2.1	Retinol dehydrogenase 7
Bg020080	2.8	3.3	2.8	AIG protein, partial
Bg019044	2.3	2.6	2.5	Galactoside 2-alpha-L-fucosyltransferase 1, partial
Bg021808	2	3.9	3.8	Persulfide dioxygenase ETHE1 mitochondrial-like isoform X1

The *S. mansoni*-susceptible RIL108_S line exhibited only a limited initial response, with 12 URGs and 50 DRGs at 6 hpe. This increased to 241 URGs and 303 DRGs at 24 hpe, and further to 1,968 URGs and 1,083 DRGs at 48 hpe (**Figures 3**, **4**). At 48 hpe, the URGs included relatively few FREPs, TEPs, FReDs, catalase, C-type lectins, GST, gram-negative bacteria-binding proteins (GNBP), lysozyme, hemagglutinin/amebocyte aggregation factor, lysosomal components, PGRP, TLRs, and aranetoxins. Conversely, the DRGs substantially overlapped with functional categories represented among the URGs of the resistant RIL18_R line, including LAAOs, IAPs, numerous FREPs, CREPs, TEPs, GiMAPs, GSTs, NF-κB inhibitors, SOD, TLRs, and dual oxidase (DUOX) (**Table 4**; **Supplementary Tables 10**, **11**).

#### 3.2.3 Shared expression profiles of resistant and susceptible lines following schistosome exposure

Comparison of the two resistant lines (iBS90_R and RIL18_R) and the two susceptible lines (iM line_S and RIL108_S) revealed remarkably few identical differentially expressed genes (DEGs) shared between lines of the same phenotype at corresponding time points following *S. mansoni* exposure. Using a stringent threshold (adjusted P < 0.01), only one gene encoding a perlucin-like C-type lectin (PLL; Bg017736) was consistently up-regulated at three time points in both resistant lines, whereas a single hypothetical protein gene (Bg025981) was consistently down-regulated at three time points in both susceptible lines (**Figure 6**). When the threshold was relaxed to adjusted P < 0.05, two additional genes, an EGF-containing protein (Bg031232) and GiMAP (Bg030694), were shared between the resistant lines, while a collectin (C-type lectin) transcript (Bg020474) was shared between the susceptible lines (**Table 5**).

**Figure 6:**
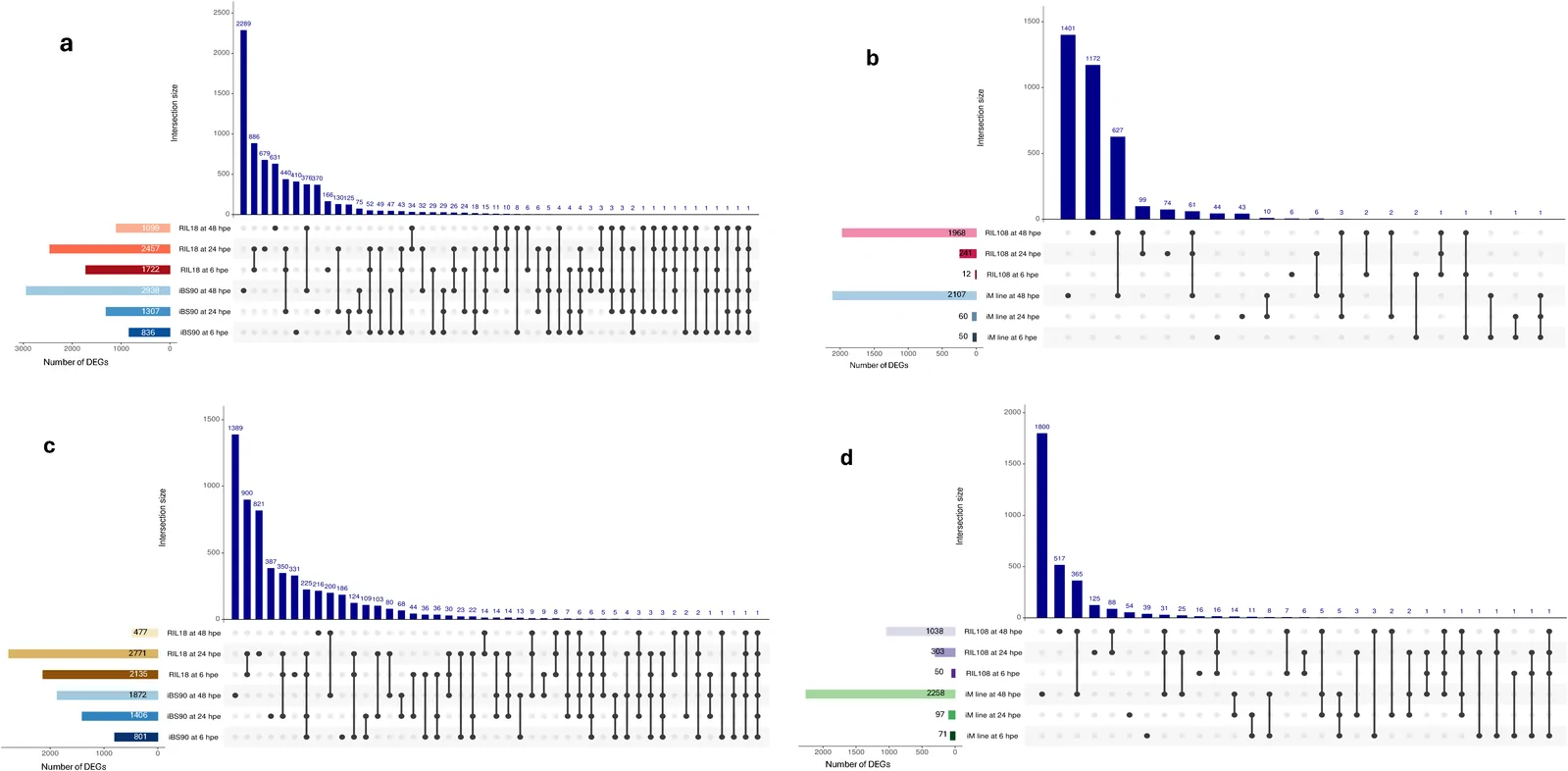
Upset plots showing shared up-regulated and down-regulated genes across all time points for snails with the same phenotype following exposure to *S. mansoni*. Diagrams **(a, b)** show up-regulated genes in resistant lines (iBS90_R and RIL18_R) and susceptible lines (iM line_S and RIL108_S), respectively, while **(c, d)** display down-regulated genes in resistant lines (iBS90_R and RIL18_R) and susceptible lines (iM line_S and RIL108_S), respectively. hpe: hours post exposure.

**Table 5:** Shared up- or down-regulated genes between iBS90 and RIL18 across all three post-exposure time points (p-adj <0.05).

		iBS90			RIL18		
		Fold change					
Gene ID	Gene product	6 hpe	24 hpe	48 hpe	6 hpe	24 hpe	48 hpe
Up-regulated gene							
Bg017736	Perlucin-like lectin (PLL)	15.22	14.49	7.73	45.67	204.13	192.91
Bg031232	Multiple epidermal growth factor-like domain containing protein	4.26	2.72	2.83	3.46	3.88	2.02
Bg030694	GiMAP	2.66	12.75	8.57	2.64	7.82	3.1
Down-regulated gene							
Bg025981	Hypothetical protein	3.7	4.61	7.71	3.55	8.48	3.93
Bg020474	Collectin	3.23	5.69	3.24	4.22	9.23	2.89

Among the shared resistance-associated genes, a PLL (Bg017736) exhibited the strongest induction. Expression increased most in RIL18_R, reaching a 204-fold increase at 24 h post-exposure (hpe), whereas induction in iBS90_R was more moderate (approximately 15-fold) (**Table 5**). Sequence analysis predicted a signal peptide, indicating that PLL is a secreted C-type lectin containing a calcium-dependent carbohydrate-recognition domain characteristic of proteins involved in pathogen recognition, binding, and immune activation (**Figure 7a**). The second shared up-regulated transcript encoded a protein containing ten tandem epidermal growth factor (EGF) domains together with a predicted signal peptide, consistent with secretion (**Figure 7b**). The third encoded a GiMAP family protein containing an AIG domain but lacking a predicted signal peptide (**Figure 7c**).

**Figure 7:**
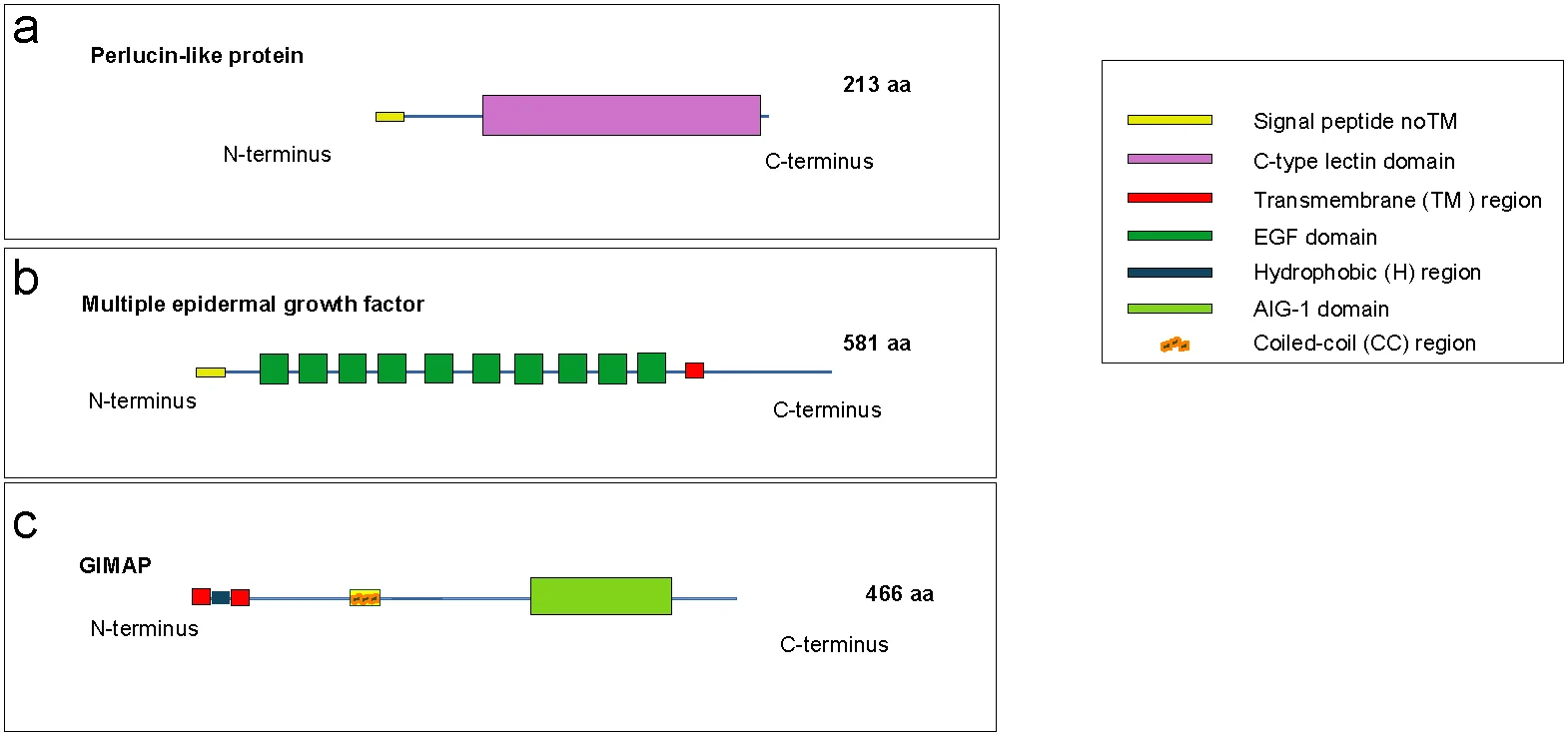
Schematic diagram showing the predicted domain structure of **(a)** Perlucin-like protein (PLL), a C-type lectin characterized by an N-terminal signal peptide and a calcium-dependent C-type lectin domain, indicating a secreted protein with function likely related to pathogen recognition; **(b)** EGF-like domain-containing protein, predicted as a transmembrane protein with extracellular EGF-like domains involved in cell signaling and adhesion; and **(c)** GiMAP protein, showing two C-terminal transmembrane helices, a coiled-coil region, and an AIG1 GTPase domain, associated with immune regulation.

Although the resistant lines shared relatively few identical DEGs, they exhibited striking convergence at the level of immune gene families. Both lines showed strong induction of FREPs, biomphalysins, and TEPs, although the specific paralogs or family members that were differentially expressed often differed between lines. In contrast, expression of these immune gene families declined by 48 hpe in both susceptible lines, with reductions in transcript abundance and/or expression levels (**Supplementary Tables 4**-**11**).

### 3.3 Gene co-expression network analysis of schistosome-resistant lines

#### 3.3.1 Identification of co-expression modules

Separate weighted gene co-expression network analyses (WGCNA) were performed on schistosome-induced differential gene expression in resistant lines in both HZL and RIL models. Soft-thresholding powers of β = 20 (HZL) and β = 22 (RIL) met the criterion for approximate scale-free topology (R² ≥ 0.8) (**Supplementary Figure 2a**). Hierarchical clustering based on topological overlap identified 21 co-expression modules in the HZL network and 15 modules in the RIL network (**Supplementary Figure 2b**).

#### 3.3.2 Module–trait associations in the HZL model

In the HZL dataset, a single module was strongly associated with the exposure-induced response of the resistant iBS90_R line. The green module showed a highly significant positive correlation with induced genes in the schistosome-resistant iBS90_R snails (R = 0.77, p = 3.68 × 10^–26^) (**Supplementary Figure 3a**). The 50 highest-ranked hub genes formed a tightly connected network, with module membership (kME) values ranging from 0.75 to 0.98, and gene significance values reaching 0.91 (**Supplementary Table 12**).

Functional annotation revealed that this module was dominated by genes involved in innate immune recognition and signaling, together with regulators of gene expression, membrane transport, protein turnover, metabolism, and cellular architecture. Immune-related hub genes included Toll-like receptor 4, a C-type lectin-related protein, the FREP family members BpFREP14.1 and BgMFREP12.3, AIG, and GiMAP7. Additional signaling components included receptor-type tyrosine phosphatases, Fyn-related kinase (FRK), MAPKK12, and cyclin-dependent kinase 20-like proteins, while regulators of transcription and chromatin organization included THO complex subunit 2, condensin-2 complex subunit D3, and nuclear transcription factor, X-box binding like 1 (NFXL1). Membrane-associated transporters, ubiquitin-mediated protein turnover, proteolytic enzymes, lipid metabolism, and cytoskeletal proteins were also well represented among the hub genes (**Figure 8**).

**Figure 8:**
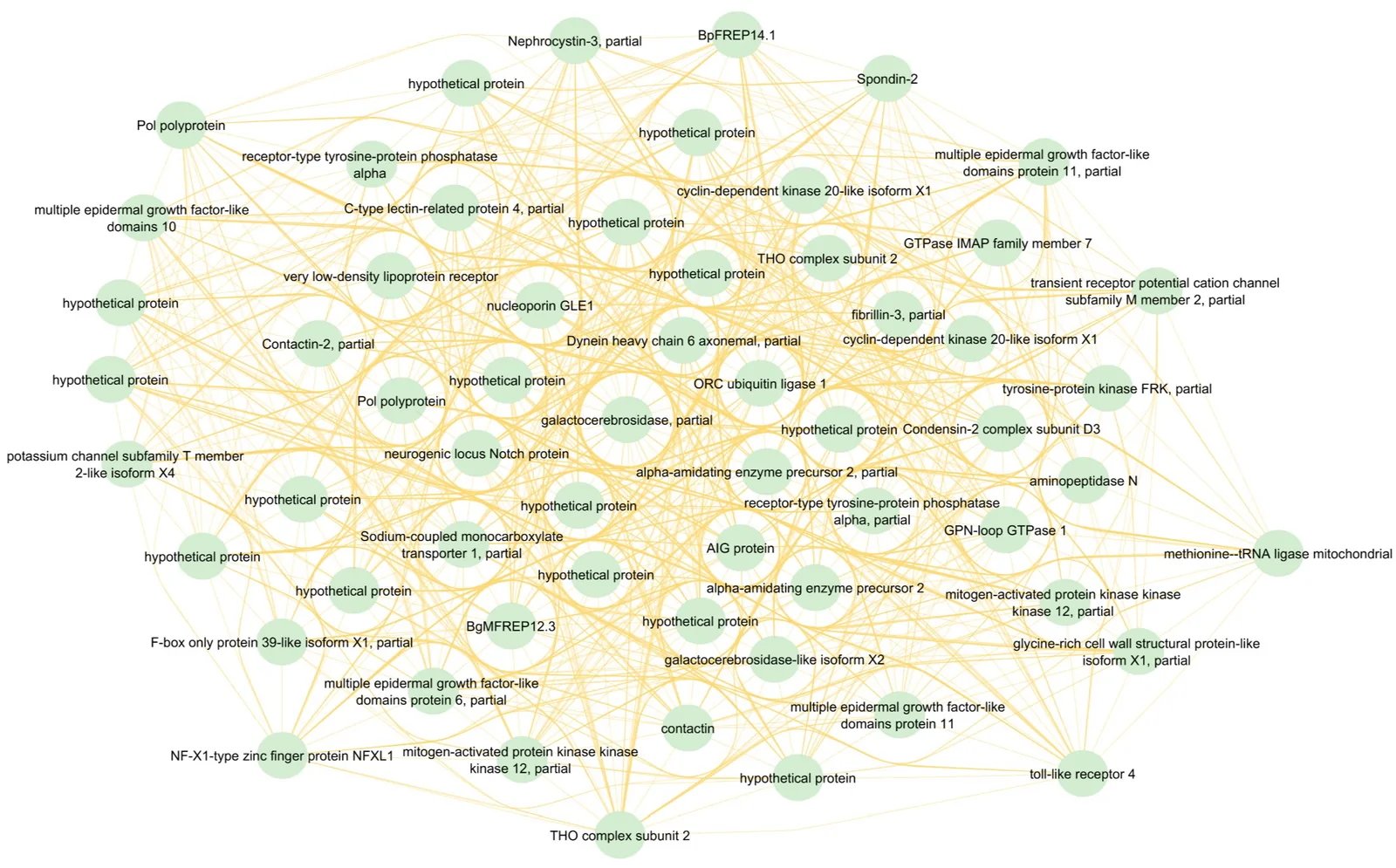
Network visualization of the top 50 hub genes from the green module associated with the resistant strain iBS90 in the HZL model. Nodes represent genes, and lines indicate intramodular connections (edges) among hub genes.

Several highly connected transcripts annotated as hypothetical protein genes were identified within the green module, suggesting that previously uncharacterized genes are also induced in the resistant snails. Overall, the exposure-induced gene module in resistant HZL snails was characterized by a network centered on immune recognition and activation, supported by signaling pathways and downstream cellular regulatory processes.

#### 3.3.3 Module–trait associations in the RIL model

In the RIL dataset, one module showed significantly induced genes in the resistant RIL18_R snails. The red module exhibited a strong positive correlation with induced gene expression in resistant snails (R = 0.70, p = 9.50 × 10^–7^) (**Supplementary Figure 3b**). The hub genes formed a highly interconnected co-expression network (**Supplementary Table 13**).

Different from the HZL network, the RIL red module was enriched predominantly for genes involved in transcriptional regulation, RNA processing, chromatin organization, protein homeostasis, mitochondrial function, and cytoskeletal organization. Prominent hub genes included multiple zinc finger transcription factors together with EHMT1, REST corepressor 3, SNF2L2, chromatin assembly factor 1, and claspin-like protein, indicating extensive regulation of gene expression and genome organization. Genes involved in ribosome biogenesis, RNA processing, and translation, including eIF3J, NHP2-like protein 1, nucleolin, nucleolar proteins, SRP68, and aminoacyl tRNA synthetase complex-interacting multifunctional protein 1, were also highly represented. The module further included genes associated with protein quality control and stress responses, such as COP9 signalosome subunit 8, ubiquitin-processing enzymes, DDI1, DnaJ family proteins, and FK506-binding proteins, together with mitochondrial metabolic enzymes and structural proteins involved in cytoskeletal organization. Several transcripts annotated as hypothetical proteins were also among the highly connected hubs (**Figure 9**). Collectively, these results indicate that the RIL18_R response is characterized by extensive transcriptional and post-transcriptional regulation, coordinated with protein homeostasis and cellular remodeling, rather than by enrichment of canonical immune recognition genes.

**Figure 9:**
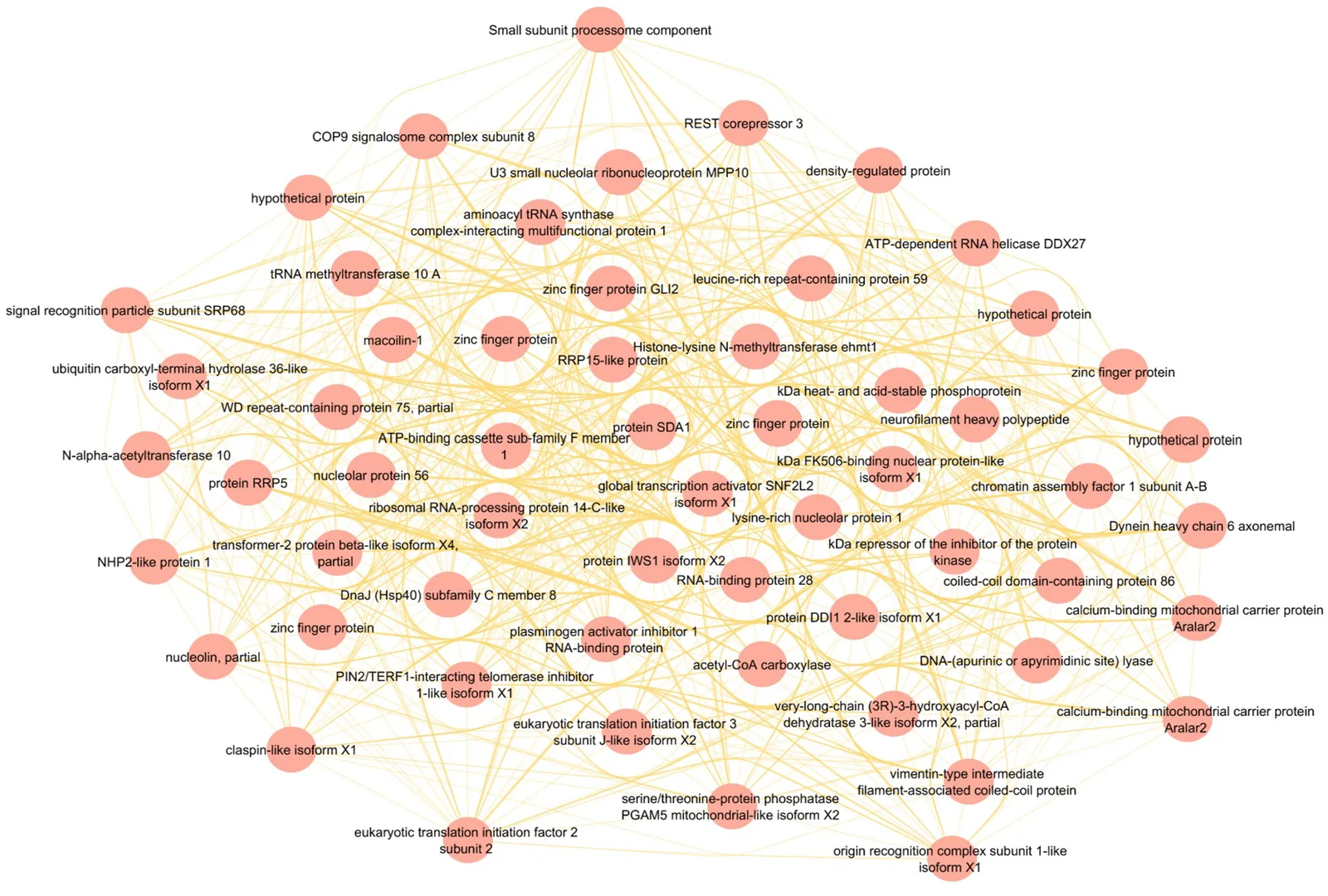
Network visualization of the top 50 hub genes from the red module that are induced in RIL18 in the RIL model. Nodes correspond to hub genes, and edges represent co-expression relationships within the module.

#### 3.3.4 Comparison of exposure-induced co-expression networks

Although both co-expression networks for induced genes in the resistant snail lines (iBS90 and RIL18) were characterized by a single co-expression module strongly associated with exposure-induced gene expression, the biological composition of these modules differed substantially. The iBS90-associated module was dominated by genes involved in pathogen recognition and innate immune signaling, including Toll-like receptors, lectins, FREPs, and GiMAPs. However, the RIL18-associated module was primarily enriched with genes involved in transcriptional regulation, RNA metabolism, chromatin remodeling, and protein homeostasis.

Despite these different network architectures, both modules contained highly interconnected hub genes involved in cellular signaling, metabolic regulation, and structural organization, suggesting that the induction of these genes may contribute to resistance, which is achieved through the coordinated activation of multiple biological processes. These findings suggest that variable transcriptional programs can converge in these resistant snail lines, with the HZL model emphasizing early immune recognition and the RIL model relying more heavily on extensive downstream regulatory mechanisms.

## 4 Discussion

### 4.1 Unique snail genetic resources provide novel insights into genome-wide gene expression in response to schistosome infection

By employing two models of well-characterized resistant and susceptible snail lines, this study leverages two complementary and previously unavailable genetic resources to perform a comparative analysis of the transcriptomic mechanisms underlying resistance and susceptibility of *B. glabrata* to *S. mansoni*. Unlike previous transcriptomic studies, which have largely relied on single models derived from natural populations or conventional laboratory strains with substantial genetic heterogeneity (13, 29, 37, 40, 62), the present study employs two pedigree-related experimental models (HZL and RIL) comprising four homozygous or highly inbred genetic lines (iBS90_R, iM line_S, RIL18_R, and RIL108_S) with highly consistent infection phenotypes (42–44). The availability of these genetically defined lines, together with their contrasting resistance and susceptibility phenotypes, provides a unique opportunity to distinguish transcriptional programs that are conserved across genetic backgrounds from those that are genotype specific.

The high level of genetic uniformity within each line minimizes biological variation among individuals, thereby increasing statistical power for genome-wide transcriptomic analyses and improving the reliability of transcriptomic comparisons. Although numerous immune genes and pathways associated with resistance to *S. mansoni* have been previously identified, as described in the Introduction, it has remained unclear whether these represent universal determinants of resistance or strain-specific responses. Moreover, discrepancies among studies, even those involving the same laboratory strains, have often been difficult to interpret because they may arise from biological differences, genetic heterogeneity within strains (63), or variation in experimental and analytical methodologies.

By integrating simultaneous comparative analysis of constitutive and schistosome-induced transcriptomic profiling with gene co-expression network analysis across two related but genetically distinct resistant–susceptible models, this framework enables the identification of both conserved transcriptional signatures shared across resistant genetic backgrounds and lineage-specific regulatory mechanisms, providing a systems-level view of the transcriptomic architecture underlying resistance.

### 4.2 Constitutive transcriptional states differ in resistant and susceptible snails

The first insight from this systems-level comparative analysis is that constitutive differential expression profiles revealed considerable transcriptional differences among all four snail lines, even between lines with the same phenotype, reflecting their distinct genetic histories and gene regulation. A variety of immune genes were among the cDEGs revealed from all four resistant and susceptible lines, aligning with the invertebrate-type innate immunity of snails that employ pre-made defense factors for pathogen recognition and immune responses (64–67). The divergence between the HZL lines was expected due to their independent origins, whereas the differences among the RIL lines demonstrate that recombination and selection generated new genomic combinations capable of producing either resistant or susceptible phenotypes.

Importantly, the resistant phenotype was associated with constitutive expression differences from the susceptible phenotype involving immune-related genes. Resistant lines exhibited increased representation of specific immune factors, including HREP (a type of VIgL) (68), NOS (Bayne et al., 2001) (69), PGRP (24), the antimicrobial aranetoxin (37, 70) and unique expression of two GiMAP family members found only in the resistant lines (37). Biomphalysin 11 (18) and a poly(ADP-ribose) polymerase also emerged as potential biomarkers of resistance in unexposed *B. glabrata*. These constitutive differences suggest that resistance is not simply the result of a stronger response after infection but instead reflects a pre-existing transcriptional state that enables more rapid and effective recognition of invading parasites.

The observation that all snail lines expressed diverse immune genes also provides an important perspective on susceptibility. Susceptible snails are not immunologically deficient; rather, they possess broad innate immune capabilities that are nevertheless insufficient against compatible *S. mansoni*. The critical difference appears to involve the regulation, timing, and coordination of immune pathways required for effective parasite recognition and elimination.

### 4.3 Resistant and susceptible snails follow different transcriptional trajectories after infection

Following parasite exposure, both resistant lines initiated rapid and extensive transcriptional responses prior to 6 hpe, the earliest time point examined, consistent with previous observations that resistant *B. glabrata* respond transcriptionally as early as 2 h after exposure (29) and routinely eliminate *S. mansoni* within 3–4 days after infection (8, 71). With increasing time post-exposure, the sDEG profiles of both iBS90_R and RIL18_R expanded to include immune factors previously shown to be instrumental in resistance to *S. mansoni*, including elevated expression of FREPs, TEPs, and biomphalysins (9, 17, 39, 72). The temporal dynamics of DEG abundance suggest that RIL18_R snails establish anti-schistosome responses more rapidly than iBS90_R. These observations indicate that functional convergence on a resistance phenotype does not necessarily require conservation of individual gene sequences. Instead, different members of multigene families appear capable of contributing to similar biological outcomes, suggesting that resistance depends on coordinated activation of immune pathways rather than any single resistance factor.

The sDEG profiles were diverse, with both resistant lines sharing only a few identical transcript sequences that exhibited elevated expression at all time points, specifically a perlucin-like lectin (PLL; a C-type lectin), a multiple epidermal growth factor domain-containing protein, and a GiMAP family member. The identification of genes/transcripts that encode PLL, a multiple EGF domain-containing protein, and a GiMAP family member in both resistant lines provides candidates for future functional investigation. PLL is a consistently induced defense-associated transcript in both resistant lines across all three post-exposure time points, even under the stringent adjusted P-value threshold (<0.01), highlighting PLL as a robust candidate involved in anti-schistosome immunity. While PLL has been implicated in immune defense in other animal taxa (73–75), its involvement in *Biomphalaria* immunity, especially anti-schistosome defense, has not been previously documented. Similarly, the gene containing multiple EGF domain sequences is identified here for the first time as strongly associated with the response to schistosome infection. EGF-like proteins may facilitate specific protein-protein interactions, mediate cell signaling, and stimulate hemocyte proliferation and differentiation during the defense response (76, 77). GiMAP family members have repeatedly been identified in snail transcriptomic studies of schistosome infection (35, 46, 78, 79); however, their functional roles remain experimentally unverified. TRPM also deserves attention, as it consistently showed the highest expression levels across three exposure time points. TRPM belongs to a family of ion channels and plays diverse functional roles, including the regulation of oxidative stress, reactive oxygen species, cell proliferation and differentiation, metabolic regulation, and homeostasis (80, 81).

The susceptible lines, however, showed relatively modest responses with fewer DEGs at 6 and 24 hpe. At 48 hpe, however, both iM line_S and RIL108_S developed expression profiles representing broad immune functions (including some FREPs, LAAOs, LBP/BPI, granulin, GRC genes, laccase, and others), while simultaneously down-regulating many transcripts that characterize the resistant response, including numerous FREPs, TEPs, biomphalysins, and other immune-related genes (see Results).

Based on the contrasting temporal patterns (immediate versus delayed responses) and differences in the representation of anti-schistosome immune factors between resistant and susceptible lines, these observations support the hypothesis that *S. mansoni* initially escapes or avoids immune recognition in susceptible snails, thereby preventing early activation of effective immune responses. Once infection becomes established, the parasite may suppress anti-schistosome defenses (e.g., down-regulation of many FREPs), while redirecting host immune activity toward broader responses against other potential pathogens. This phenomenon may represent a general feature of compatible digenetic trematode-snail associations. A transcriptomic study of *Echinostoma paraensei* infection in susceptible *Physella acuta* proposed a similar scenario in which the infected snail effectively becomes an extended phenotype of the parasite (82). Conversely, the immune recognition systems of both resistant *B. glabrata* lines appear capable of detecting invading *S. mansoni* immediately, thereby rapidly activating immune genes required for parasite elimination. Differences in the specific gene sequences involved and in the speed of response likely reflect the distinct genomic architectures of the two resistant lines.

A better understanding of the mechanistic determinants of resistant and susceptible phenotypes in the *S. mansoni–B. glabrata* interaction will require further investigation of how parasite-derived molecules interact with snail recognition systems that regulate immune gene expression, as well as how parasites modify the expression of key immune effectors such as FREPs, TEPs, biomphalysins, and the resistance-associated genes identified here, including PLL, GiMAP, and EGF-domain-containing proteins. Several additional studies support the importance of parasite-mediated immune modulation. *Schistosoma mansoni* induces epigenetic changes in *B. glabrata* that may influence susceptibility (83, 84), and, like other digenetic trematodes, releases products during infection that modulate snail immune responses (85–87).

### 4.4 Different gene regulatory networks are involved in resistance in two resistant lines

The gene co-expression network analyses provided strong indications that resistance represents a systems-level property rather than the activity of individual immune genes, which is generally consistent with schistosome-induced expression profiles as discussed above. Despite sharing a common resistant phenotype, the HZL and RIL resistant snails exhibited markedly different exposure-associated network architectures.

In the HZL model, the exposure-associated module was dominated by genes involved in innate immune recognition and signaling, including Toll-like receptors, C-type lectins, FREPs, immune-associated GTPases, and signaling components. This network structure is consistent with rapid recognition of invading parasites and activation of downstream immune effector pathways. In contrast, the exposure-associated module in the RIL model was enriched for genes involved in transcriptional regulation, chromatin organization, RNA processing, protein homeostasis, and cellular remodeling. This alternative organization suggests that resistance in the recombinant genetic background is achieved through greater reliance on transcriptional regulation, chromatin remodeling, and cellular reprogramming. The difference between the two resistant networks is particularly notable because RIL18_R originated from the resistant iBS90_R parental background. Despite this shared ancestry, extensive recombination generated a distinct regulatory architecture that nevertheless maintained the resistant lines.

Collectively, these findings indicate that resistance can arise through multiple molecular routes. Rather than requiring a single conserved regulatory network or immune gene repertoire, resistance may emerge through coordinated regulation of diverse biological processes. The involvement in a co-expression network of numerous genes without previously assigned immune functions further emphasizes that successful parasite elimination depends on the integration of immune recognition, cellular physiology, metabolism, and regulatory pathways.

## 5 Conclusion

A systems-level comparative analysis of the HZL and RIL genetic models provides novel insights into compatibility between *B. glabrata* and *S. mansoni*. The major contribution of this study is not simply the identification of additional immune-associated transcripts and hypothetical genes, but the demonstration that genetically defined snail resources can provide new insights into fundamental questions regarding the molecular architecture of resistance. Although this study suggests that regulation of snail global gene expression in response to schistosome infection is much more complicated than previously thought, three principal conclusions emerge from this comparative analysis. First, resistance is associated with distinct constitutive transcriptional states that distinguish resistant from susceptible snails before infection occurs. Second, resistant snails rapidly activate immune pathways following parasite exposure, but different genetic backgrounds accomplish this through different combinations of genes and regulatory programs. Third, susceptibility is not caused by the absence of immune capability but is associated with delayed and altered regulation of immune pathways that may be influenced by parasite-mediated modulation. Thus, resistance to *S. mansoni* in *B. glabrata* is best understood as a genetically heterogeneous but functionally convergent phenotype. Different *B. glabrata* lineages can employ distinct transcriptional architectures and regulatory networks while converging on overlapping biological processes required for effective parasite elimination. These findings provide a broader framework for understanding the compatibility between schistosomes and their snail hosts and identify candidate pathways for future functional investigation.

## Data Availability

The data supporting the findings of this study are included within the article and its supplementary information files. Raw RNAseq data are available in the NCBI Sequence Read Archive database under BioProject accession number PRJNA1390394.

## Supplementary material

Supplementary Figure S1 Principal component analysis (PCA) of RNAseq-based gene expression profiles showing transcriptomic variation among four snail genotypes (iM line, iBS90, RIL18, and RIL108) across 0, 6, 24, and 48 hpe, with PC1 and PC2 explaining 82% and 14% of the total variance, respectively.

Supplementary Figure S2 Soft-thresholding power selection for WGCNA was performed separately for HZL and RIL. A: HZL model. a) Scale-free topology model fit (signed R²) across candidate β values; β = 20 was selected at R² ≥ 0.8. b) Mean connectivity versus β, supporting β = 20 as a compromise between scale-free fit and network connectivity. B: RIL model. c) Scale-free topology model fit (signed R²); β = 22 satisfied the R² ≥ 0.8 criterion. d) Mean connectivity versus β, supporting β = 22 for network construction.

Supplementary Figure S3 Module–trait correlation heatmaps for the a) HZL and b) RIL models. Each cell represents the correlation between a module eigengene and an experimental trait, where color indicates the strength and direction of association, and numbers indicate correlation coefficients. Statistical significance is indicated as follows: p < 0.05 *, p < 0.01 **, p < 0.001 ***.

Supplementary Table 1 Summary of quality assessment results for 78 RNAseq read datasets used in gene expression analyses.

Supplementary Table 2 Constitutively differentially expressed (pre-exposure) genes in homozygous inbred lines of *B. glabrata*, restricted to genes with an expression ratio ≥ 10 and assigned functional annotations. a) Resistant inbred line iBS90 relative to susceptible inbred line iM. b) Susceptible inbred line iM relative to resistant inbred line iBS90.

Supplementary Table 3 Constitutively differentially expressed (pre-exposure) genes in recombinant inbred lines of *B. glabrata*, restricted to genes with an expression ratio ≥ 10 and assigned functional annotations. a) Resistant recombinant inbred line RIL18 relative to susceptible recombinant inbred line RIL108. b) Susceptible recombinant inbred line RIL108 relative to resistant recombinant inbred line RIL18.

Supplementary Table 4 Differentially up-regulated genes in the homozygous resistant inbred line *B. glabrata* iBS90 following *S. mansoni* exposure at a) 6 hours post-exposure (hpe), b) 24 hpe, and c) 48 hpe.

Supplementary Table 5 Differentially down-regulated genes in the homozygous resistant line *B. glabrata* iBS90 following *S. mansoni* exposure at a) 6 hpe, b) 24 hpe, and c) 48 hpe.

Supplementary Table 6 Differentially up-regulated genes in the homozygous susceptible line *B. glabrata* iM line following *S. mansoni* exposure at a) 6 hpe, b) 24 hpe, and c) 48 hpe.

Supplementary Table 7 Differentially down-regulated genes in the homozygous susceptible line *B. glabrata* iM line following *S. mansoni* exposure at a) 6 hpe, b) 24 hpe, and c) 48 hpe.

Supplementary Table 8 Differentially up-regulated genes in the recombinant resistant line *B. glabrata* RIL18 following *S. mansoni* exposure at a) 6 hpe, b) 24 hpe, and c) 48 hpe.

Supplementary Table 9 Differentially down-regulated genes in the recombinant resistant inbred line *B. glabrata* RIL18 following *S. mansoni* exposure at a) 6 hpe, b) 24 hpe, and c) 48 hpe.

Supplementary Table 10 Differentially up-regulated genes in the recombinant susceptible inbred line *B. glabrata* RIL108 following *S. mansoni* exposure at: a) 6 hpe, b) 24 hpe, c) 48 hpe.

Supplementary Table 11 Differentially down-regulated genes in the recombinant susceptible inbred line *B. glabrata* RIL108 following *S. mansoni* exposure at a) 6 hpe, b) 24 hpe, and c) 48 hpe.

Supplementary Table 12 Hub genes associated with phenotypes in the HZL model. Top 50 hub genes from the green module associated with the resistant iBS90 phenotype. Columns: Module (co-expression module assignment); kME (correlation between the gene and the module eigengene); kME_p (p-value of kME); kWithin (intramodular connectivity); GS (gene significance for iBS90).

Supplementary Table 13 Hub genes associated with phenotypes in the RIL model. Top 50 hub genes from the red module associated with the resistant RIL18 phenotype. Columns: Module (co-expression module assignment); kME (correlation between the gene and the module eigengene); kME_p (p-value of kME); kWithin (intramodular connectivity); GS (gene significance for RIL18).
